# Green Hydrophobic Modification of Biomass-Derived Porous Carbon: Linking Surface Chemistry, Pore Structure, and Sustainability

**DOI:** 10.3390/molecules31203684

**Published:** 2026-10-14

**Authors:** Xiaohong Wang, Jingxin Wang

**Affiliations:** 1 School of Art and Media, Guangzhou Vocational University of Science and Technology, Guangzhou 510550, China; wangxiaohong@gkd.edu.cn; 2 Guangdong Provincial Engineering Research Center of Public Health Detection and Assessment, School of Public Health, Guangdong Pharmaceutical University, Guangzhou 510310, China

**Keywords:** biomass-derived porous carbon, hydrophobic modification, surface-confined covalent esterification, green chemistry, paradigm shift

## Abstract

The hydrophobic modification of biomass-based porous carbon involves two theoretical paradigms—“exogenous coating” and “surface-confined covalent transformation”—but the underlying logical transition between them has not yet been systematically examined. This study employs a theoretical dialectical approach. Through the deconstruction of the concept of “green hydrophobic modification,” the deduction of progressive propositions, and a critical dialogue with existing research, we have constructed a three-dimensional integrated analytical framework comprising “chemical logic—spatial effects—green attributes.” The study found that the efficacy of hydrophobic modification is constrained by the synergistic contradiction between the degree of surface chemical transformation and the preservation of pore structure; exogenous modifications, such as silanization and fluorination, tend to fall into a “hydrophobicity–pore” trade-off whose severity depends on the modifier molecular size, degree of functionalization, grafting density, pore size distribution, and alkyl chain length, due to the coverage by a heterogeneous hydrophobic layer, whereas surface-confined covalent esterification based on esterification with natural organic acids directly converts oxygen-containing functional groups on the carbon surface into ester groups, achieving logical consistency between hydrophobic functionality and pore structure preservation. The study further reveals that the surface-confined covalent esterification paradigm holds a significant advantage in terms of alignment with green chemistry principles, though its applicability is limited by the abundance of surface functional groups on the carbon precursor. These findings provide clear criteria for evaluating paradigms in the green hydrophobic modification of biomass-derived porous carbon, propelling related research from methodological description toward theoretical construction. A three-way classification of modification routes is introduced (exogenous coating; exogenous reagent + surface-confined covalent transformation; truly endogenous transformation), and the verification requirements (solid-state NMR/XPS), pore-sensitive wettability metrics, and hydrolytic-stability limits of surface-confined covalent esterification are explicitly delimited.

## 1. Introduction

Research on biomass-based porous carbon materials is rooted at the intersection of green chemistry and sustainable materials science, with its theoretical origins traceable to the dual academic traditions of activated carbon surface chemistry and pore engineering. Early research focused on the development of high specific surface areas and well-developed pore structures, attributing improvements in adsorption performance to the optimization of physical structure. However, with the advancement of interfacial science, theories of surface wettability have gradually been introduced into the field of porous carbon research, leading to a divergence between two major theoretical perspectives: “structure-dominated” and “interface-regulated.” The former emphasizes the decisive role of pore size and specific surface area, while the latter asserts that surface chemical properties govern the adsorption-material interaction mechanisms. The mechanistic basis of the “interface-regulated” perspective was consolidated by the systematic classification of carbon surface functionalities in classical studies of activated carbon surface chemistry, which established that the identity and density of surface oxygen groups can be quantified reproducibly and deliberately tuned by oxidative or thermal treatments [1,2].

This theoretical divide has become increasingly pronounced in practical applications within the field of environmental water treatment: when porous carbon materials are required to selectively adsorb hydrophobic organic pollutants from aqueous solutions, their inherent hydrophilic nature becomes a key bottleneck limiting adsorption efficiency. Existing research has shown that the surfaces of unmodified biomass charcoal are rich in polar functional groups such as carboxyl and hydroxyl groups [3,4]. While these chemical properties facilitate dispersion in water, they weaken the material’s affinity for hydrophobic organic molecules, thereby sparking academic interest in the deeper theoretical issue of “synergistic regulation of structure and surface properties.” Comparable conclusions have been drawn for the broader family of biochars: the dynamic molecular structure of plant biomass-derived black carbon comprises condensed aromatic domains coexisting with a significant density of oxygenated functionalities [5], and the relative abundance of these polar groups directly governs the affinity of biochar for hydrophobic organic contaminants such as phenanthrene and bisphenol A [6].

The scope of biochar’s application as an environmental remediation material is rapidly expanding. A 2024 review in Biomass and Bioenergy by Lan et al. synthesized progress in biochar modification technologies for environmental remediation across soil, water, and atmospheric systems from 2018 to 2023 [7]. It clearly identified that, among the three major categories of methods—chemical, physical, and biological modification—“enhancing the number of oxygen-containing functional groups, increasing micropore content, and improving stability through modification” are key directions. This review provides a broad application context for this paper’s focus on the chemical modification pathway of “surface-confined covalent esterification of natural organic acids.” Comprehensive reviews have likewise established biochar as a low-cost and sustainable sorbent platform for contaminant management in soil and water [8], and the development of biochar-based functional materials is increasingly framed as a route toward a sustainable platform carbon material that preserves the renewable character of the biomass precursor [9].

Currently, research on the hydrophobic modification of biomass-based porous carbon faces three theoretical controversies and gaps. First, regarding the intrinsic logic of modification pathways, while traditional methods—such as silane coupling agents and chemical fluorination—can create hydrophobic surfaces, their theoretical basis relies on the physical coating or chemical grafting of exogenous hydrophobic groups. This process essentially severs the connection between the inherent chemical properties of biomass materials and surface restructuring, resulting in the theoretical tension between “green raw materials” and “chemical modification” not being adequately addressed. Second, regarding the explanation of the structure-property relationship of modification effects, existing studies have primarily focused on macroscopic wettability indicators such as contact angle, while the mechanisms by which hydrophobic modification affects the chemical heterogeneity of internal surfaces within multi-level pore channels have not yet been fully elucidated theoretically [10,11]. In particular, thermodynamic and kinetic models for wettability transitions within microporous confined spaces urgently need refinement. Chen et al. (2026) systematically reviewed research progress on the adsorption of VOCs by hydrophobic-modified activated carbon under high-humidity conditions, focusing on the principles, modification effects, and research examples of five major hydrophobic modification strategies: organic molecular surface modification, halogen atom doping, metal oxide loading, organosilane coating, and high-temperature heat treatment, thereby providing the latest literature foundation for the comparative analysis in this paper [11]. Third, regarding the completeness of the theoretical framework, natural organic acid modification—as an emerging approach—achieves surface-confined covalent hydrophobization through mild esterification reactions. However, no studies have yet conducted a systematic comparison or reflection on whether this mechanism can theoretically transcend the traditional exogenous modification paradigm [12,13,14].

This leads to the core theoretical question of this study: From silane fluorination to natural organic acids, has the intrinsic logic of hydrophobic modification of biomass-derived porous carbon undergone a paradigm shift from “exogenous hydrophobization” to “surface-confined covalent restructuring”? How does this shift theoretically bridge the gap between “structural regulation” and “interface design”?

The theoretical value of this study lies in its attempt to provide clear theoretical boundaries for the concept of “green hydrophobicity” through a systematic review and critical comparison of the chemical mechanisms and structure-property relationships of different hydrophobic modification pathways. The study helps clarify the dialectical relationship between “modification” and “preservation of green properties,” offering a new perspective for the surface engineering of porous carbon materials—shifting from “methodological superposition” toward “theoretical integration”—and addressing the current shortfall in the field, which tends to prioritize the optimization of preparation processes while neglecting an examination of the intrinsic logical underpinnings of modification theory. It provides a theoretical reference for advancing the rational design of biomass-derived carbon materials in the field of selective adsorption.

The main text will be structured around the analytical framework of “conceptual deconstruction → comparative analysis of approaches → logical reflection → theoretical integration.” First, we will deconstruct the chemical essence and evaluation dimensions of hydrophobic modification; next, we will analyze the logic behind exogenous modification via silane fluorination; subsequently, we will critically examine the surface-confined covalent esterification theory underlying modification with natural organic acids; and finally, through a comparison of these two paradigms, we will examine the theoretical implementation pathways of green chemistry principles in the surface engineering of porous carbon.

## 2. Concept Deconstruction of Hydrophobic Modification

In the process of adsorption functionalization of biomass-derived porous carbon, the term “hydrophobic modification” has evolved from a simple surface treatment process into a theoretical framework that embodies the philosophy of materials design and the values of green chemistry. To thoroughly examine the paradigm shift from silane fluorination to natural organic acids, we must first systematically deconstruct this core concept, clarifying its essential attributes, functional boundaries, and shifts in meaning across different academic contexts.

In terms of its essential attributes, the hydrophobic modification of porous carbon is not merely a simple surface treatment to repel water, but rather the integration of a three-step process: “chemical modification—interface restructuring—property transformation.” First, any hydrophobic modification begins with chemical modification—that is, through covalent grafting, noncovalent coating, or surface functional group conversion—to transform the originally abundant polar oxygen-containing groups (such as hydroxyl and carboxyl groups) into nonpolar or weakly polar groups with low surface energy. Research by Zhao et al. (2026) demonstrated that porous charcoal derived from corn stover, prepared via thermal activation with zinc chloride, is rich in functional groups such as carboxyl, hydroxyl, and carbonyl groups, with an oxygen content ranging from 5.7% to 9.0% [3]. This composition constitutes the source of its hydrophilicity and serves as the chemical site for subsequent hydrophobic modification. Second, chemical modification inevitably leads to interfacial restructuring, namely the formation of a molecular layer with low surface energy characteristics on the inner walls of the pores, thereby altering the interaction patterns between water molecules and the carbon walls. This interfacial restructuring not only involves an increase in the macroscopic contact angle but also alters the clustering behavior of water molecules within the confined spaces of micropores and mesopores. Alatalo (2016) pointed out that the hydrothermal carbonization process can increase the material’s aromaticity and endow it with a hydrophobic framework; this suggests that the establishment of hydrophobicity can result either from the introduction of chemical groups or from the depolarization of the carbon framework’s own structure [10]. Third, these changes ultimately culminate in a transformation of the material’s properties: shifting from the original aqueous dispersion and high water absorption to a state that preferentially adsorbs organic hydrophobic molecules and repels water molecules. Therefore, the hydrophobic modification of porous carbon is, in essence, a chemical process that intentionally rewrites the hydrophilic “chemical memory” inherited from biomass into a hydrophobic “surface identity.” The implications of this concept must be understood dynamically as the causal link between modification methods, interfacial chemical states, and macroscopic wetting behavior; it cannot be simplified to mere increases or decreases in contact angle values.

**Figure 1:**
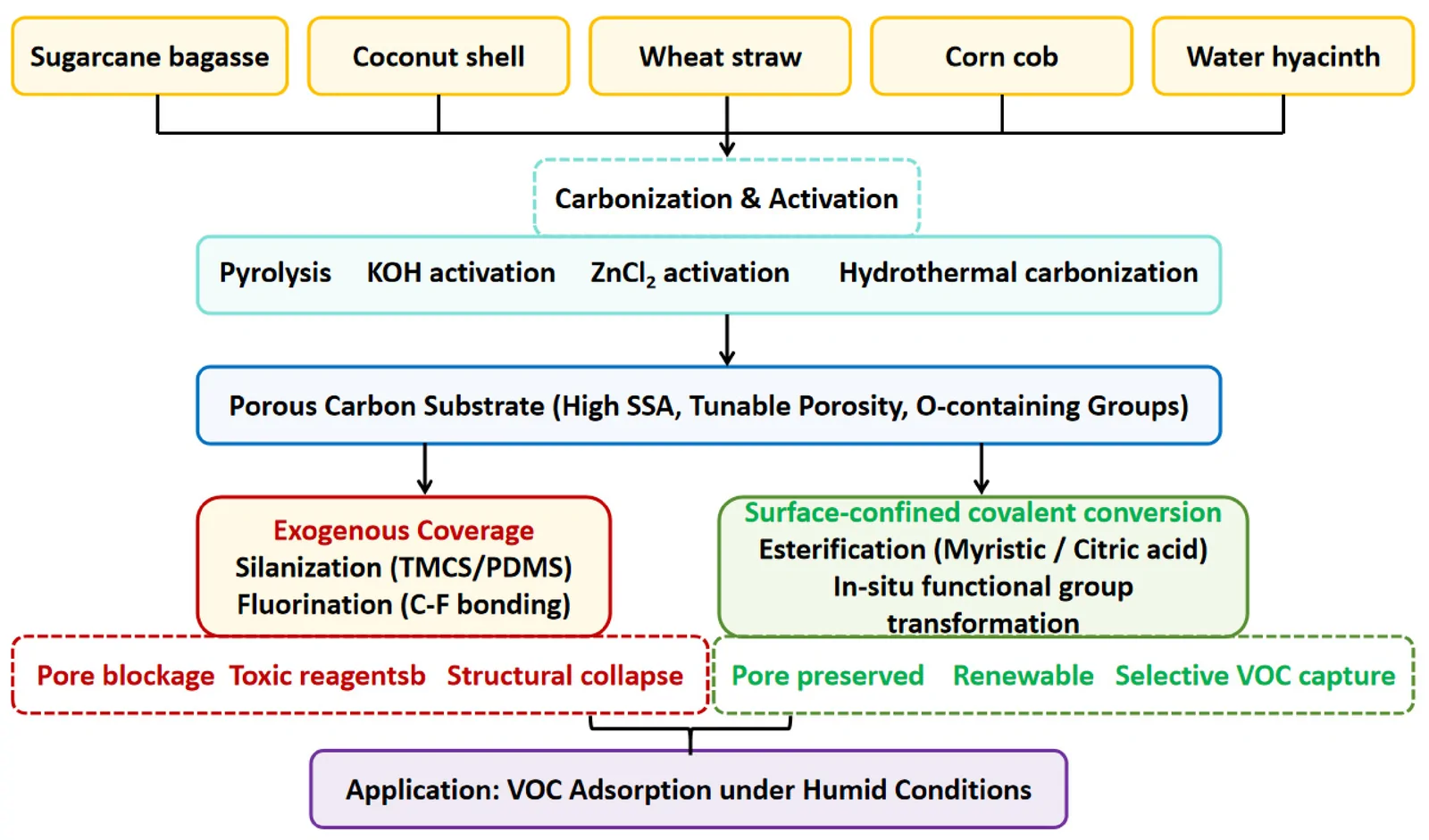
Schematic illustration of biomass-derived porous carbon fabrication and modification strategies for VOC adsorption. (**a**) Porous carbon substrates are prepared from biomass via carbonization and activation. (**b**) Two modification pathways: exogenous coverage (e.g., silanization) and surface-confined covalent transformation (e.g., esterification). (**c**) The surface-confined covalent esterification strategy offers superior VOC capture performance under humid conditions.

As shown in Figure 1, biomass precursors (such as sugarcane bagasse, coconut shells, wheat straw, corn cobs, and water hyacinth) are carbonized and activated to form a porous carbon matrix, after which they follow one of two distinct pathways: exogenous coating (silylation/fluorination) and surface-confined covalent transformation (esterification with natural organic acids). These two pathways exhibit systematic differences across three dimensions—chemical logic, spatial effects, and green attributes—and form the structural foundation for the subsequent line of reasoning in this paper.

From a functional perspective, the core objective of hydrophobic modification is the targeted enhancement of selective adsorption performance. In environmental remediation scenarios, although unmodified biomass porous carbon can achieve a specific surface area of 712.1–1667.5 m^2^ g^−1^ and possesses a rich network of multi-level pores [3], when confronted with weakly polar or nonpolar pollutants in water—such as phenol and chlorinated organics—its strong surface hydrophilicity causes water molecules to form a hydrogen-bond network on the carbon surface, competing for sites and creating a water film barrier that hinders the diffusion of organic molecules into the pore channels. Work on carbon electrodes for supercapacitors likewise shows that the surface chemistry of activated carbon directly determines ion transport and adsorption affinity; while hydrophilicity facilitates wetting by aqueous electrolytes, it may limit selectivity toward specific ions. Therefore, the purpose of hydrophobic modification is to weaken the competitive adsorption between water molecules and the carbon surface through surface depolarization, thereby enhancing the thermodynamic affinity of the carbon material for organic pollutants [15]. Hamimed and Chatti (2024) explicitly pointed out that surface modification of porous carbon can transform it from hydrophilic to hydrophobic, thereby enhancing the removal efficiency of specific pollutants [16]. This implies that the concept of “hydrophobic modification” incorporates a dimension of functional expectation: it does not aim for absolute, complete hydrophobicity, but rather adopts selective polarity matching as a design criterion, seeking the optimal range on the hydrophilic–hydrophobic continuum. This functional characteristic distinguishes hydrophobic modification from purely structural modifications (such as pore size expansion); while the latter also influences adsorption, its mechanism of action involves improvements in physical transport rather than the targeted regulation of surface thermodynamics. It should also be noted that specific surface area and pore size distribution must be interpreted within the standardized physisorption methodology codified in the IUPAC technical report on the evaluation of surface area and pore size distribution [17], and that the adsorption of organic molecules from aqueous solutions on carbon materials is jointly governed by pore structure and surface chemistry rather than by surface area alone [18].

From a value-oriented perspective, the selection of hydrophobic modification methods increasingly reflects the profound penetration of green chemistry principles into materials science. Traditional surface hydrophobization strategies, such as silane coupling agent treatment and fluoride grafting, can efficiently reduce surface energy and create hydrophobic or even superhydrophobic interfaces. However, as noted by several scholars, these methods rely on synthetic chemicals such as fluorinated compounds or organosilanes, and their preparation processes, environmental fate after use, and biocompatibility all raise significant sustainability concerns [12]. In contrast, the approach of modifying materials with natural organic acids, which has gained traction in recent years, seeks to re-anchor hydrophobic modification engineering in the context of renewable resources and non-toxic, harmless reagents. Jha et al. (2021) point out that the sustainability of surface-modified activated carbon should be incorporated into evaluation systems; true sustainability requires that the functionalization of carbon materials not come at the expense of the original eco-friendly properties of biomass [12]. These studies have collectively spurred a trend toward reevaluating values: hydrophobic modification is no longer merely a matter of technical specifications, but rather a value judgment regarding “what kind of modification can be defined as green.” The reason this study adopts “green hydrophobic modification” as its core concept is precisely to define a system of modification pathways that both achieves hydrophobic functionality and adheres to the boundaries of green chemistry—it requires compliance with the basic principles of sustainable development across dimensions such as the origin of modification reagents, the mildness of reaction conditions, the toxicity of byproducts, and the environmental compatibility of modified materials throughout their entire life cycle. In this way, the concept of hydrophobic modification embodies the dual value orientations of “functional requirements” and “ecological responsibility.” These considerations directly echo the foundational formulation of green chemistry—designing chemical products and processes that reduce or eliminate the use and generation of hazardous substances [19]—its subsequent articulation as a research frontier spanning feedstock selection, reaction conditions, and product life cycle [20], and the quantitative E factor concept, which exposes the waste burden of reagent-intensive modification routes and thereby sharpens the comparison between exogenous and surface-confined strategies [21].

Looking back at the historical evolution of this concept, two intertwined threads can be clearly identified. In the early stages, the concept of modifying porous carbon was closely linked to the “surface chemical regulation” of activated carbon, with its theoretical foundation rooted in the adsorption theories that emerged from the activated carbon industry in the late nineteenth to early twentieth centuries. With the development of surface analysis techniques such as IR and XPS, functional groups such as carboxyl, lactone, and phenolic hydroxyl groups were identified one by one, and researchers began to consciously modulate surface polarity through methods such as oxidation, reduction, and heat treatment. After the 1990s, inspired by research on biomimetic superhydrophobic surfaces and self-assembled monolayers, silanization and fluorination treatments were introduced into the field of porous carbon, forming a modification paradigm dominated by “exogenous hydrophobic agents.” However, with the rise of the green chemistry movement, this paradigm has come under scrutiny. In particular, the research by Yuan et al. (2022) on constructing microporous biochar through citrate esterification, as well as the patent by Wang et al. (2021) on biochar modified with natural organic acids, clearly pointed to a shift in approach from exogenous chemical grafting to surface-confined covalent esterification reactions, marking a significant green transformation of the concept of “hydrophobic modification” [13,14]. It is evident that the theoretical evolution of this concept has not been a linear accumulation but rather a spiral ascent between “efficiency-oriented” and “green-oriented” approaches. The adsorption theories of the activated carbon industry referred to above are systematically compiled in the classical monographs on activated carbon production, structure, and adsorption [22,23].

Based on this, it is necessary to distinguish several concepts that are easily confused with “green hydrophobic modification.” First, a distinction should be made between “surface hydrophobization” and “bulk hydrophobicity.” The former refers solely to changes in the chemical properties of the surface of carbon material pores, while the latter may involve the introduction of a hydrophobic carbon coating or an increase in the overall degree of carbonization, resulting in the material becoming hydrophobic as a whole. The adsorption capacity of porous carbon relies primarily on surface chemistry; therefore, this study focuses on surface hydrophobization rather than framework hydrophobicity achieved by increasing the degree of graphitization, which is often accompanied by a loss of specific surface area. Second, it is necessary to distinguish the relationship between “physical activation” and “chemical modification.” Physical activation (such as steam or CO_2_ activation) can, in itself, remove some oxygen-containing functional groups and enhance hydrophobicity; this constitutes a form of “passive hydrophobization.” Chemical modification, on the other hand, involves the active introduction of specific functional groups or reagents and is classified as “active hydrophobization.” The one-step pyrolysis method employed by Yang et al. (2023) in the preparation of nitrogen-phosphorus-sulfur co-doped porous carbon combines both carbon framework formation and surface heteroatom regulation [4]; however, the surface chemical changes involved in this process extend beyond the scope of physical activation. Furthermore, the distinction between “exogenous modification” and “surface-confined covalent esterification” is crucial. Traditional methods such as silane fluorination are typical examples of exogenous modification, i.e., the introduction of a heterogeneous hydrophobic layer from the outside; whereas the esterification reaction between natural organic acids and the hydroxyl groups inherent in the biomass itself constitutes surface-confined covalent esterification—that is, surface restructuring achieved by grafting natural hydrophobic groups using the reactive sites present within the material itself. This distinction serves as the central theoretical starting point for this study’s critique: the essence of green hydrophobic modification requires a shift in the modification logic from exogenous physical or chemical coating to surface-confined covalent transformation, thereby maximizing the preservation of the material’s intrinsic green properties. Finally, the scope of the qualifier “green” must be clarified. Jha et al. (2021) point out that while strong acid oxidation can remove mineral components and increase surface hydrophilicity, it may also introduce sulfur- or nitro-containing byproducts [12]. Therefore, the requirements of green chemistry must permeate the entire modification process—including the use of non-toxic or low-toxicity reagents, mild reaction conditions, and biodegradable waste—and the hydrophobic effect of the final modified product cannot serve as the sole criterion for evaluation.

Three-way classification of modification routes: (i) exogenous coating/deposition, in which the modifier is physically attached to the surface without covalent bonding; (ii) exogenous reagent + surface-confined covalent transformation, in which an externally supplied molecule reacts covalently with hydroxyl or carboxyl groups that belong to the carbon precursor itself—the category to which natural organic acid esterification belongs; and (iii) truly endogenous transformation, relying solely on the precursor’s own constituents. The term “endogenous” is no longer used for routes employing externally supplied reagents; throughout the revised text it has been replaced by “surface-confined covalent esterification”, with “intrinsic oxygen functionalities” used when referring specifically to the native –OH/–COOH groups of the precursor.

To deconstruct and apply the above concepts to the specific context of this study, it is necessary to adapt the concept of “green hydrophobic modification” to this context. This study focuses on biomass-based porous carbon, whose precursors include agricultural and forestry waste such as corn stover and Chinese fir sawdust. Its structural characteristic lies in the retention of a large number of oxygen-containing functional groups resulting from the depolymerization of lignocellulose. This chemical memory provides natural reaction sites for the esterification grafting of natural organic acids. In terms of modification methodology, this means that “green” is no longer an external label but is intrinsically coupled with the chemical structure of the biomass itself. Specifically, natural polycarboxylic compounds—such as citric acid, tartaric acid, and gallic acid—can react with hydroxyl groups on the surface of the carbon through mild thermal condensation or catalytic esterification, thereby constructing a hybrid surface on the inner walls of the pores that features hydrophobic hydrocarbon chains and residual polar anchoring points. This modification method not only derives its reagents from renewable resources but also, in its reaction mechanism, represents an extension of biomass chemistry rather than a break from it. Therefore, this study defines “green hydrophobic modification” as follows: based on the inherent oxygen-containing functional groups of biomass-derived porous carbon, using natural, renewable organic acids as modifiers, and through mild esterification or condensation reactions—while preserving the integrity of the material’s porous structure and the tunability of its surface chemistry—achieving a functional transformation from a hydrophilic to a hydrophobic state, and ensuring that the entire modification pathway meets the requirements of green chemistry regarding atomic economy and low environmental risk. This definition integrates intrinsic properties (chemical esterification and interfacial restructuring), functional characteristics (enhanced selective adsorption), and value orientation (sustainability), thereby establishing a logical benchmark for subsequent paradigm comparisons.

## 3. Exogenous Modification: Mechanism and the Apparent “Hydrophobicity–Pore” Trade-Off

Building on the multidimensional deconstruction of the core concept of “green hydrophobic modification” presented in the previous section, this section will develop a logical argument through propositional deduction, focusing on the clues to a paradigm shift inherent within the concept. If the task of the previous section was to define clear connotations and boundaries for the concept, then the mission of this section is to reveal the structural causal relationships and inherent tensions among these constituent elements. We must address the following questions: Why do exogenous modifications, such as silane fluorination, fall into a “hydrophobicity–porosity” trade-off, and under which parameter combinations does this trade-off become severe? And how does surface-confined covalent esterification based on natural organic acids theoretically constitute a logically consistent transcendence of this dilemma? The esterification route is taken up in Section 4, where the representative natural organic acids and their reported esterification performance are compiled in Table 1. To answer these questions, we cannot merely provide a list-style description of different modification methods; rather, we must delve into the microscopic mechanisms of the interaction between surface chemistry and pore structure, constructing a logical chain that starts from fundamental propositions, proceeds through progressive derivation, and culminates in a dialectical synthesis.

**Proposition 1.**  *The efficacy of hydrophobic modification is governed by the synergy between “surface chemical transformation” and “pore structure preservation.” An inherent structural tension exists between these two factors, defining a fundamental trade-off in the engineering of porous carbon.*

This proposition is rooted in the most fundamental performance requirements for porous carbon materials as adsorbents. The efficiency of any adsorption process depends simultaneously on two conditions: first, whether the adsorbate molecules can diffuse smoothly within the pore channels and come into contact with the pore wall surfaces—that is, the mass transfer kinetics; second, whether the pore walls can exert sufficient thermodynamic affinity for the adsorbate molecules—that is, the adsorption equilibrium conditions. In biomass-derived porous carbon, mass transfer hinges on the integrity and connectivity of its multi-scale pore structure. The study by Zhao et al. (2026) strongly demonstrates this [3]: The porous carbon material prepared from corn stover as the feedstock and zinc chloride as the activator exhibited excellent phenol adsorption performance precisely because its well-developed multi-level pore system—comprising micropores, mesopores, and macropores—and its high specific surface area ranging from 712.1 to 1667.5 m^2^ g^−1^ provided ample space for the diffusion and accommodation of adsorbate molecules. At the same time, the surface chemical state represents the other key factor determining affinity. Yang et al. (2023) observed during the preparation of nitrogen-phosphorus-sulfur co-doped porous carbon that the introduction of surface heteroatoms significantly altered the material’s electrochemical properties and ion adsorption behavior, indicating that the type and density of surface chemical groups directly influence the interactions between the carbon material and target molecules [4].

From this, a fundamental contradiction can be derived: the goal of hydrophobic modification is to transform the carbon surface from a highly polar, hydrophilic state to a low-polarity, hydrophobic state, which necessarily requires the introduction of nonpolar or weakly polar groups. However, these introduced groups—whether present via covalent grafting or noncovalent coating—tend to occupy space within the pore channels—the extent of the resulting geometric disturbance depending on modifier size, degree of functionalization, and grafting density—altering the original geometric morphology of the pore walls. In other words, increasing the degree of chemical modification and preserving the pore structure constitute a pair of conflicting factors that must be balanced in terms of physical space and chemical mechanisms. Any modification strategy that unilaterally pursues performance in one aspect may result in substantial, difficult-to-reverse losses in another dimension, thereby ultimately weakening the material’s overall adsorption efficiency. This proposition establishes the fundamental theoretical context for all subsequent analyses: the merits of hydrophobic modification cannot be determined by a single indicator such as contact angle, but must be evaluated within the dynamic balance between “chemical conversion” and “structural preservation.”

Parameter-dependence of the “hydrophobicity–pore” trade-off: The severity of the trade-off between hydrophobicity gain and porosity loss is not fixed but is governed by identifiable parameters: the molecular size of the modifier; the degree of functionalization; the grafting density; the pore size distribution of the substrate (particularly the fraction of ultramicropores, whose apertures approach the kinetic diameter of the grafted moiety); and the alkyl chain length, which determines both the hydrophobic contribution per graft and the conformational footprint of the grafted layer. Small, sparsely grafted modifiers may leave microporosity largely intact, whereas bulky, densely grafted layers progressively narrow pore mouths and may ultimately block them. Accordingly, all previously absolute formulations in this review (“inevitably fall into”, “irreversible pore blockage”, “structural paradox”) have been rewritten as condition- and parameter-dependent statements. As emphasised in recent critical reviews of VOC adsorption and surface-modification methods [24], the balance between surface-chemistry enhancement and textural degradation is a central design variable rather than a fixed penalty; comparative oxidation studies likewise show that the extent of textural damage is strongly treatment-dependent [25].

**Proposition 2.**  *Traditional external modification relies on the deposition of a heterogeneous hydrophobic layer. This “coating rather than transformation” mechanism essentially triggers an apparent “hydrophobicity–porosity” trade-off, whereby enhanced water repellency tends to come at the cost of (partially) irreversible pore constriction or blockage—the extent of which depends on modifier size, grafting density, and pore size distribution—and a reduction in specific surface area.*

Building on the logical premise of Proposition 1, we may further ask: What mechanistic strategies do different modification approaches employ to address the tension between surface chemical conversion and pore structure preservation? And what structural consequences do these strategies entail? A fundamental characteristic of the exogenous modification paradigm is that the modification reagents it employs—whether long-chain alkylsilanes, fluorinated silane coupling agents, or perfluorocarboxylic acids—lack chemical homology with the biomass carbon framework. These reagents are forcibly “loaded” onto the carbon surface through exogenous physical or chemical means. In this process, the acquisition of hydrophobicity primarily relies on two mechanisms: first, the formation of one or more densely packed layers of hydrophobic molecular films on the inner walls of the pores, which reduce surface energy by covering the original hydrophilic sites; second, the formation of a chemically bonded hydrophobic “brush layer” on the surface, which alters wettability by introducing long-chain alkyl or fluorocarbon chains. For example, Kaneko et al. [26] confirmed through N_2_ adsorption and XPS that fluorination modification reduced the pore width of activated carbon fibers by 0.21 nm, and that N_2_ adsorption within the fluorinated micropores was significantly reduced due to diffusion limitations; Mussabek et al. [27] further found that the microporous structure shrinks during fluorination at temperatures above 400 °C: after treatment at 600 °C, the micropore area fell from 1156 to 815 m^2^ g^−1^ and the BET area from 1254 to 902–941 m^2^ g^−1^; this finding directly confirms the negative impact of excessive surface modification on pore transport capacity. One further observation is warranted at this point. No study could be located in which a fluorination or fluoroalkylation treatment was applied to a biomass-derived porous carbon with both textural and uptake data reported; the fluorination evidence cited above rests on activated carbon fibres [26] and on a coal-derived commercial carbon [27]. This absence is worth stating explicitly rather than leaving as a gap in Table 2, because it is itself consistent with the trajectory argued in this review: the fluorination route is being displaced by bio-derived alternatives before a comparable body of biomass-based evidence was ever generated. It also means that any quantitative comparison of fluorination with esterification on biomass substrates is, at present, not possible—a limitation that the minimum reporting standard proposed in Section 7.4 is intended to remove. Quantitative fluorination data are available for non-biomass carbons and are discussed in Section 4, where they support the same argument: the textural cost rises steeply with the degree of fluorination rather than being an intrinsic property of the route. The mechanistic basis of silanization is well established in silane chemistry: alkylchlorosilanes hydrolyse and condense into siloxane (Si–O–Si) networks whose growth competes directly with the accessibility of the underlying pores [28], while the density of reactive surface hydroxyl sites available for condensation governs the thickness and heterogeneity of the resulting grafted layer [29].

Guo et al. (2021) conducted dynamic competitive adsorption studies at 0%, 50%, and 90% humidity using custom-blended siloxane-functionalized activated carbon [30]. They found that although siloxane loading reduced the equilibrium water absorption from 21.9% to 7.2% and increased the contact angle by 70.10°, theoretical calculations of adsorption energy simultaneously confirmed that: the siloxane layer only marginally enhances the selectivity of competitive adsorption between water molecules and VOC molecules within the micropores. This study reveals, at the molecular scale, the fundamental reason why the exogenous coating paradigm cannot thoroughly resolve the “hydrophobic-pore” trade-off. In the field of adsorption, this finding carries more direct physical implications. Taking typical fluorination modification as an example, the cross-sectional area of fluorocarbon segments is far greater than that of water molecules or common small organic molecules. When the inner wall of a microporous channel is densely coated with fluorocarbon chains, its effective pore diameter shrinks dramatically and may even be completely blocked. Similarly, when silane coupling agents undergo hydrolysis and condensation on the carbon surface, they often tend to form cross-linked siloxane networks. Such networks not only occupy space but may also block adjacent micropores in a chain-like manner, causing the originally accessible active surface to become inoperative due to the “gateway effect.” Chandrasekaran et al. (2017) pointed out that carbon aerogels possess a large specific surface area and a through-pore structure [31]; although they did not directly discuss the issue of blockage, their overall observations on the relationship between pore structure and wettability point to an indisputable fact: the introduction of hydrophobicity is always accompanied by drastic changes in the chemical and physical environment within the pore channels.

Recent empirical studies have further quantified the structural cost of exogenous silanization: Li et al. [32] modified activated carbon using TMCS impregnation, which increased the contact angle from 111.6° to 143.6° and boosted toluene adsorption capacity by 91% at 90% RH compared to unmodified carbon; their study also confirmed that the hydrolysis-driven formation of the siloxane network occupies micropore space, providing a vivid illustration of the “hydrophobic-pore” trade-off associated with exogenous modification. In their latest review, Chen et al. (2026) reached a compatible conclusion by a different, biological route: pretreating linden wood with Ganoderma lucidum yielded a hydrophobic biochar (FTBC) without recourse to grafting chemistry, and the authors state explicitly that conventional hydrophobization strategies such as siloxane grafting involve the use and release of hazardous chemicals and therefore conflict with the principles of green chemistry [33] (that study is examined in detail in Section 4). The systematic comparison of the available strategies—organic molecular surface modification being simple but of limited hydrophobicity, whereas organosilane coating is more complex but reaches higher contact angles—is drawn from the recent review of hydrophobic modification for VOC capture under high humidity [11]. Hamimed and Chatti (2024), in their discussion of porous carbon for water treatment, further pointed out that while activation can transform hydrophilicity into hydrophobicity, the pore characteristics and surface heterogeneity of the modified carbon-based materials are significantly altered [16]. With respect to fluorination, the structural chemistry of covalent graphite fluorides, in which fluorine forms strong C–F bonds at the expense of the π system and interlayer spacing, is documented in the classical monograph on graphite fluorides [34], which accounts for both the rigidity and the chemical persistence of fluorocarbon coatings.

The study by Li et al. (2019) on PDMS-coated modified walnut shell activated carbon is particularly compelling: although the PDMS coating enabled the material to maintain good VOC adsorption performance at 90% RH, their study explicitly pointed out that this modification process “damaged the pore structure and surface functional groups of the adsorbent, consumed large amounts of chemical reagents, and caused secondary environmental pollution.” [15]. This finding is significant because it is the authors’ own assessment rather than an inference drawn here; it corroborates the limitations of the exogenous silanisation paradigm specifically in respect of reagent burden and environmental sustainability. It should not, however, be read as evidence that silanisation is intrinsically more destructive to pore structure than esterification, which the compiled data do not show (see above). The humidity-stability advantage reported in the same study—saturated uptake falling by 19.3% from RH 0 to 90%, against 55.9% for the unmodified carbon—is likewise a genuine benefit of the coating and is acknowledged as such; it simply does not offset the reagent burden or the dry-condition penalty. In stark contrast, Wang et al. [35] achieved an increase in contact angle from 54.15° to 134.80° through surface-confined covalent esterification via myristic acid (tetradecanoic acid, C14:0) esterification, while maintaining a micropore distribution similar to that of unmodified AC. On the evidence available, however, this contrast should not be pressed into a claim that silanisation damages the pore structure more severely than esterification. Where both routes have been characterised on comparable microporous carbons, the net textural losses are in fact similar: myristic-acid esterification lowered the BET area and micropore volume of a bagasse-derived carbon by 8.7% and 11.4% respectively [35], whereas the TEOS + TMCS treatment of a coconut-shell carbon lowered them by 7.8% and 7.1% [32]—marginally less. The distinction between the two paradigms therefore lies not in the magnitude of textural loss but in where the cost is incurred. For silanisation the cost is comprehensive: total pore volume falls by 18.5% because TEOS divides mesopores into micropores, a redistribution that flatters the BET figure while reducing the total void volume actually available; in dry gas the modified carbon adsorbs around 20% less toluene than the unmodified carbon, and the authors of that study state explicitly that the method is unsuitable for dry conditions; regeneration is poor, the saturated capacity falling by 36–39% over three cycles, worse than the unmodified carbon; and the reagent burden (TEOS, TMCS, n-hexane) is substantial. Esterification, by contrast, incurs its smaller textural cost without any of these penalties and with a renewable reagent [35]. We have therefore reframed the comparison from “structural preservation” to “total cost of the modification”, which is the dimension on which the two paradigms genuinely differ. Meanwhile, while the myristic acid esterification modification increased the contact angle to 134.80°, the micropore distribution remained close to that of unmodified AC—this contrast vividly illustrates the significant differences between the two paradigms of exogenous silanization and surface-confined covalent esterification along the three dimensions of “structural preservation-process simplification-performance enhancement.” Recent research has further confirmed the decisive role of structural preservation in the adsorption of high-humidity VOCs from the perspective of synergistic regulation of “pores–chemistry–interfaces.” Wei et al. (2026) prepared nitrogen-doped bamboo charcoal (BCNK2-800) via pyrolysis at 800 °C using a mass ratio of 2:1:1 for KOH, urea, and bamboo charcoal [36]. The specific surface area increased from 332.96 m^2^ g^−1^ to 1590.4 m^2^ g^−1^, and the water contact angle increased from 116.5° to 137.9°. At 80% relative humidity, the adsorption capacity for dichloromethane remained at 87.6% (535.2 mg g^−1^) of that under dry conditions. DFT calculations confirmed that nitrogen doping enhanced the adsorption energy of the carbon layer for VOCs and suppressed the competitive adsorption of water molecules. This study directly corroborates the core proposition of this paper: only by performing surface hydrophobization while preserving the multi-level pore structure can excellent adsorption performance be achieved in high-humidity environments—this is precisely the structural advantage that the exogenous coating paradigm struggles to attain, yet which the surface-confined covalent transformation paradigm inherently possesses. Consequently, the fundamental contradiction of the exogenous modification paradigm becomes clearly apparent: the hydrophobization method on which it relies is precisely the same force that destroys the porous structure; while reducing surface energy, it also physically weakens the material’s mass transfer advantages as a “porous” carbon. This is not a technical issue that can be resolved by optimizing process parameters, but rather an apparent trade-off—governed by modifier size, grafting density, and pore size distribution—inherent in the modification mechanism of “exogenous heterogeneous layer formation” itself.

## 4. Surface-Confined Covalent Esterification: Paradigm Shift

**Proposition 3.**  *Surface-confined covalent esterification utilizes inherent oxygen-containing functional groups to graft hydrophobic groups. By directly integrating hydrophobic functionality into the carbon matrix, this strategy largely resolves the structural trade-off and achieves a logically consistent balance between high hydrophobicity and structural integrity.*

The dilemma of exogenous modification revealed by Proposition 2 provides a clear direction for logically seeking an alternative paradigm: Is there a modification strategy that can reduce surface polarity while maximizing the preservation of the geometric integrity of the porous structure? The surface-confined covalent esterification paradigm is the response to this logical inquiry. Its chemical basis lies in the fact that biomass-derived porous carbons—especially those prepared via medium- to low-temperature pyrolysis or chemical activation—naturally carry abundant oxygen-containing functional groups on their surfaces. These functional groups are not “defects” to be masked, but rather valuable resources capable of undergoing chemical transformation.

**Figure 2:**
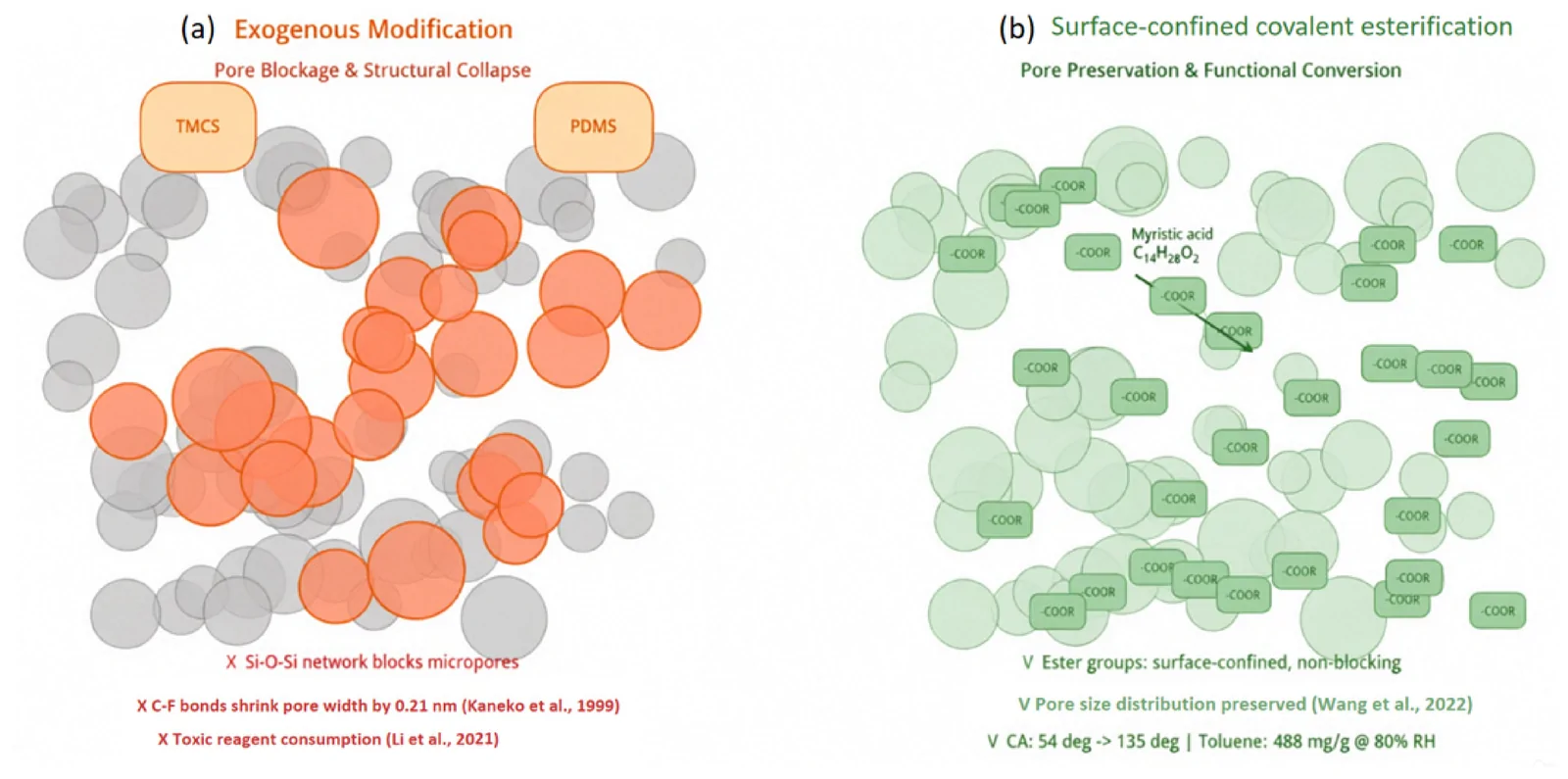
Comparative illustration of the microstructure and hydrophobic modification mechanisms. (**a**) Exogenous coating (silanization with TMCS/PDMS, or fluorination) deposits a heteroepitaxial hydrophobic layer that occupies pore space; the extent of pore narrowing depends on modifier size, grafting density and pore-size distribution and, on comparable microporous carbons, is not necessarily larger than that of the esterification route (Table 2). (**b**) Surface-confined covalent esterification (e.g., myristic acid, C14:0; citric acid) converts native –OH/–COOH groups into surface ester (–COOR) groups in situ, anchoring the graft at the atomic scale rather than depositing a separate layer, so that the pore cross-section remains essentially unchanged. The two paradigms therefore differ in the total cost of modification rather than in the magnitude of pore loss alone (Section 3). The two panels are distinguished by colour and shape: in (**a**) the orange-red coating is drawn as coarse, block-like bodies that fill and constrict the pore channels, whereas in (**b**) the green –COOR groups are drawn as thin motifs confined to the pore wall. Sources: Kaneko et al. (1999) [26] and Li et al. (2021) [32] for the mechanistic features in (**a**); Wang et al. (2022) [35] for the contact-angle and toluene-uptake data in (**b**).

Figure 2 provides a visual comparison of the structural consequences of the two modification paradigms. The left panel (exogenous modification) shows silane/fluoride coatings accumulating as coarse, block-like structures on the surface of the carbon framework and within the pore channels, leading to a drastic reduction in the effective pore diameter of the micropores; the right side (surface-confined covalent esterification) shows ester groups (–COOR) anchored compactly to the carbon surface without occupying pore space. This visual comparison directly validates the core assertion of Proposition 2: exogenous coating encounters the “hydrophobic-pore” trade-off due to the formation of a heterogeneous layer, whereas surface-confined covalent transformation achieves logical consistency through in situ functional group transformation.

Zhao et al. (2026) reported that the oxygen content of porous carbon ranged from 5.7% to 9.0%, and the doped carbon studied by Yang et al. (2023) similarly possessed abundant surface heteroatoms [3,4]. These data reveal an important fact: the surface of biomass carbon inherently possesses sufficient sites to undergo chemical reactions with natural organic acids. Natural organic acids, such as citric acid, tartaric acid, and gallic acid, contain both carboxyl or anhydride groups in their molecular structures—which can react with hydroxyl or phenolic hydroxyl groups on the carbon surface—and relatively short hydrocarbon backbones or aromatic rings, which serve as sources of hydrophobicity. This esterification mechanism has been supported by empirical evidence. In their study on citric acid-modified water hyacinth biochar, Xu et al. (2016) used FTIR, XPS, and other techniques to provided evidence consistent with citric acid undergoes an esterification reaction with hydroxyl groups on the biochar surface to form new ester bonds, resulting in a significant increase in the adsorption capacity of the modified biochar for methylene blue [37]. This study confirmed the feasibility of the “surface-confined covalent esterification” pathway from a chemical reaction mechanism perspective—the formation of ester bonds allows hydrophobic hydrocarbon chains to become a natural extension of the carbon surface, rather than a heterogeneous coating. The underlying organic chemistry has been firmly established in the textile literature on cellulose esterification: polycarboxylic acids such as citric acid esterify hydroxyl-bearing surfaces through the intermediate formation of five-membered cyclic anhydrides, and only those acids capable of forming such anhydride intermediates act as effective esterifying agents [38].

When citric acid undergoes an esterification reaction with the hydroxyl groups on the carbon surface, the result is the formation of an ester bond and the grafting of a hydrophobic side chain derived from the citric acid molecule. The key point is that this esterification reaction does not introduce a large, heterogeneous unit independent of the carbon skeleton, but rather converts the original hydrophilic functional group “in situ” into an ester group that serves both anchoring and hydrophobic functions. From a chemical structural perspective, the acyl carbon in the ester group is directly linked to an oxygen atom on the carbon surface and can be regarded as a continuation and reorganization of the carbon surface’s chemical structure, rather than an externally added coating.

Complementing the surface-confined covalent esterification pathway, recent studies have confirmed the feasibility of intrinsic hydrophobization of biochar from the perspective of “process innovation.” Additionally, Lin et al. (2024) prepared hydrophobic porous biochar through programmed microwave rapid pyrolysis (650 °C, 15 min) combined with low-dose FeCl_3_ catalysis, increasing the specific surface area from 288.6 m^2^ g^−1^ to 455.9 m^2^ g^−1^ [39]. The adsorption capacities for benzene and toluene reached 136.6 mg g^−1^ and 94.6 mg g^−1^, respectively, while the adsorption capacity for water vapor was only 6.2 mg g^−1^. This study reveals an important mechanism: the synergistic construction of “hydrophobicity and porosity” can be achieved by regulating the pyrolysis process itself (rather than through exogenous modification). This provides corroboration for the surface-confined covalent esterification paradigm from a process perspective—the establishment of hydrophobicity does not require heterogeneous coatings but can be achieved through the directed transformation of the intrinsic chemical structure of biomass. Related process innovations continue to emerge: systematic variation of feedstock and microwave-pyrolysis parameters (heating rate, activation temperature, feedstock composition) has been shown to control the porosity of the resulting biochars [40], and a synergistic nonthermal-plasma/K^+^-ion-exchange pretreatment of rice husk has recently yielded highly hydrophobic microporous biochar with a p-xylene adsorption capacity of 281.3 mg g^−1^ even at 100% relative humidity [41].

Yuan et al. (2022) introduced carboxyl groups onto the surface of biochar via citric acid esterification and ion exchange, achieving highly efficient capture of gaseous para-xylene (maximum adsorption capacity of 110.74 mg g^−1^) [14]. This study demonstrated the technical feasibility of surface functionalization of biochar using natural organic acids, which aligns with the concept of surface-confined covalent esterification modification. The study by Wang et al. (2022), published in Chemical Engineering Journal, provides the most direct empirical support for the surface-confined covalent esterification paradigm [35]. In this study, porous carbon derived from sugarcane bagasse was used as a support and hydrophobic modification was achieved through liquid-phase esterification covalent grafting with myristic acid (a medium-chain natural fatty acid (C14:0)). The contact angle of the resulting AC-MA increased from 54.15° to 134.80°; at 80% relative humidity, the toluene adsorption capacity increased significantly from 151 mg g^−1^ to 488 mg g^−1^; the toluene/water vapor selectivity predicted by IAST jumped from 11 to 150. FTIR and XPS characterization provided indicative (though not conclusive) evidence for the occurrence of the esterification reaction (formation of O–C=O bonds; see the spectroscopic-verification paragraph added in Section 4), while GCMC molecular simulations further revealed that the adsorption enthalpy of AC-MA for toluene was higher than that of unmodified AC, whereas its adsorption enthalpy for water was lower than that of unmodified AC, thereby confirming the selective adsorption advantages of surface-confined covalent esterification modification from a thermodynamic perspective.

Spectroscopic verification of surface esterification: Because a C=O band or changes in the intensity of the O–C=O feature in FTIR spectra can also arise from residual carboxylic acids, lactones, and adsorbed carboxylate species, FTIR evidence alone is treated here as indicative rather than conclusive. A multi-technique verification protocol is recommended: (i) solid-state 13C NMR, in which the ester carbonyl resonates at a chemical shift (~170–175 ppm) distinct from that of free carboxylic acid and carboxylate carbon; (ii) XPS C 1s, in which the ester O–C=O component appears at a higher binding energy (~288.5–289.0 eV) than the carboxyl/carboxylate component; and (iii) a chemical control experiment, namely base-catalysed hydrolysis of the grafted layer followed by re-measurement of contact angle and FTIR, which directly tests whether the spectral feature belongs to a hydrolysable ester. Studies relying solely on FTIR are flagged as indicative in Table 1. The identification and quantification of the relevant surface functionalities should follow the established protocols of carbon surface chemistry, beginning with selective titration schemes for carboxylic, lactonic, and phenolic groups [1] and complemented by XPS analysis of the C 1s and O 1s regions, whose sensitivity to oxidation-induced changes in surface oxygen groups is well documented [2].

Steric hindrance in confined micropores and chain-length trade-offs: Increasing the alkyl chain length of the grafting acid raises the hydrophobic contribution per graft but simultaneously increases the effective kinetic diameter and conformational footprint of the grafted moiety. Beyond a certain chain length, the marginal hydrophobicity gain is offset by pore-mouth narrowing and by the entropic penalty of confining a flexible chain, giving rise to non-monotonic behaviour. Short-to-medium chains (C12–C16, e.g., myristic acid, C14:0) generally offer the more favourable trade-off for microporous carbons, whereas longer chains (C18:0 and above) are better tolerated in mesoporous supports. This non-monotonic, chain-length-dependent behaviour is consistent with the modification–structure–performance relationships compiled in recent reviews of VOC adsorption on modified porous carbons [24].

**Figure 3:**
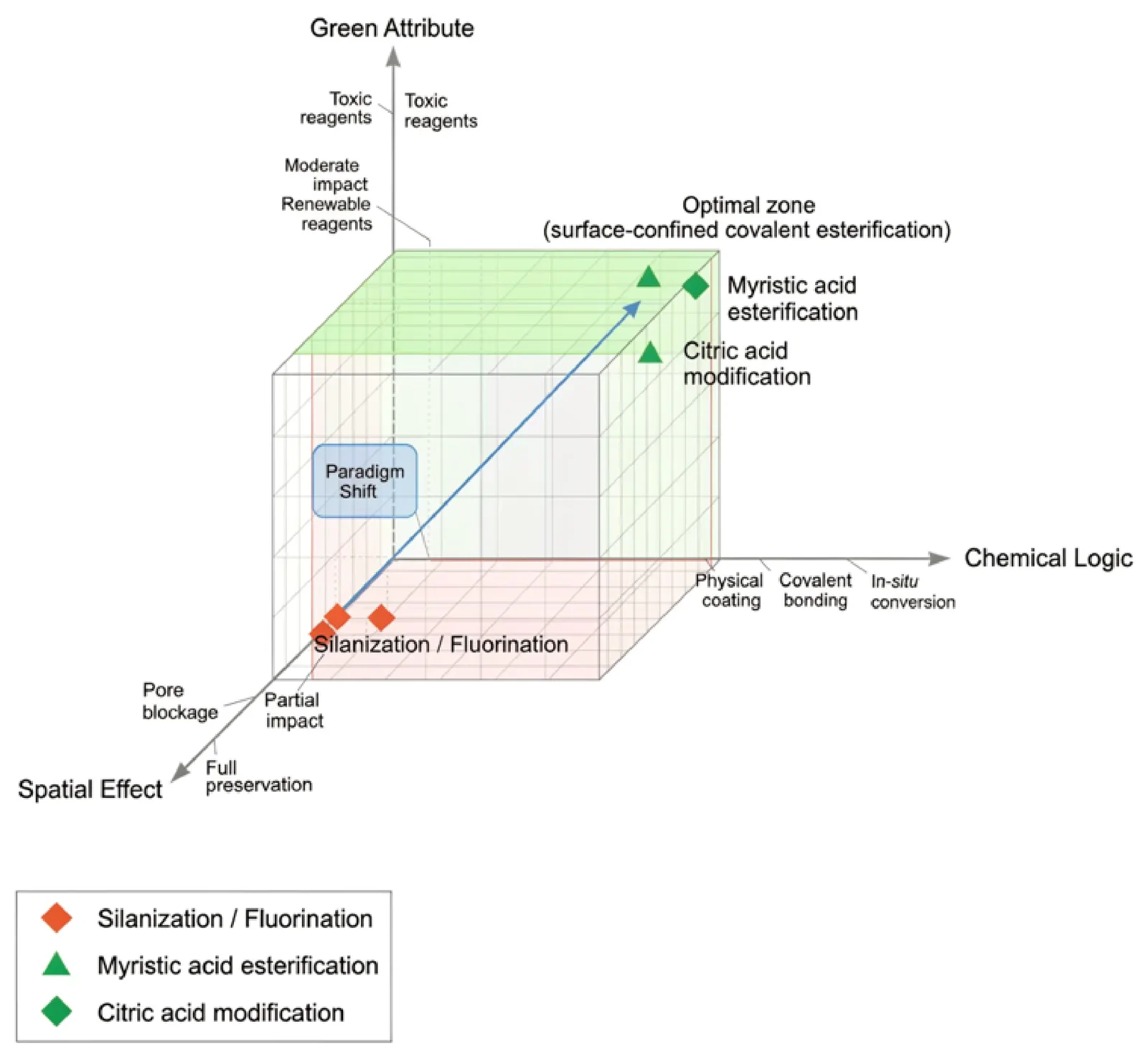
Three-dimensional integrated framework positioning hydrophobic-modification strategies on three ordinal axes: chemical logic (physical coating → covalent bonding → in situ conversion), spatial effect (pore blockage → partial → full preservation), and green attribute (toxic → moderately impactful → renewable). The green translucent volume marks the optimal zone of surface-confined covalent esterification, and the arrow indicates the paradigm shift. Marker positions are qualitative.

Figure 3 presents the integrated analytical framework proposed in this paper in three-dimensional coordinates. The X-axis represents chemical logic (from physical coating to covalent bonding to in situ conversion), the Y-axis represents spatial effects (from pore blockage to partial preservation to complete preservation), and the Z-axis represents green attributes (from toxic reagents to moderately impactful reagents to renewable reagents). The green semi-transparent region in the figure indicates the “optimal zone” (the region where the surface-confined covalent transformation paradigm resides), while the orange/red scatter points mark the cluster of exogenous modification strategies. The arrow pointing from the exogenous zone to the surface-confined covalent esterification zone symbolizes the directional force of the “paradigm shift.” Figure 3 presents this framework. Because the three axes are scored independently, a strategy’s position in the cube is diagnostic rather than merely descriptive: exogenous routes (silanisation, fluorination) cluster toward pore blockage and toxic reagents, whereas esterification with natural organic acids falls inside the optimal zone. The framework is used below to grade the routes compiled in Table 2.

Since surface-confined covalent esterification does not introduce large volumes of heterogeneous layers but instead achieves polarity reversal through chemical bond rearrangement at the atomic scale, the spatial geometry within the pore channels is disturbed far less than in exogenous modification. The morphology of the microporous walls is largely preserved, the connectivity of mesopores and macropores is not blocked by obstructions, and losses in the material’s specific surface area and pore volume are minimized. Thus, the surface-confined covalent esterification paradigm offers, for the first time, the possibility of a logical reconciliation between the once-sharp contradiction of enhancing surface chemical conversion while preserving pore structure. Precisely for this reason, surface-confined covalent esterification is not merely an alternative technical option but a method that signifies a paradigm shift—it fundamentally alters the chemical philosophy underlying the establishment of hydrophobic functions, moving from “coating” to “transformation.”

Similarly, research by Tomczyk et al. (2024) on citric acid-modified wheat straw biochar also confirmed that citric acid modification alters the surface chemistry of biochar, introducing new functional groups—such as ester groups—onto the material’s surface [42]. This finding provides cross-cultural corroboration of the surface-confined covalent esterification paradigm proposed in this paper: whether it is the Eastern team’s use of myristic acid to modify biochar [35], or the European team’s use of citric acid to modify wheat straw biochar [42], the common chemical essence is the conversion of biomass’s inherent oxygen-containing functional groups into hydrophobic ester groups via esterification reactions, thereby achieving a reversal of surface polarity without damaging the carbon framework. Table 1 summarizes the esterification effects of representative natural organic acids in the hydrophobic modification of porous biomass char. Myristic acid achieved the most comprehensive performance breakthrough (contact angle of 134.8°, toluene adsorption of 488 mg g^−1^, and IAST selectivity of 150) [35]; Citric acid has been shown to introduce ester functional groups and enhance adsorption performance in both water hyacinth biochar [37] and wheat straw biochar [42]; malic-acid-activated corncob biochar (reclassified in revision: acid-assisted activation/carbonisation, not surface esterification) [43] even achievedhydrophobic (water contact angle 120°) and an adsorption capacity of 1850 mg g^−1^. These empirical data, spanning different precursors and acid types, collectively support a core conclusion—with the reclassified acid-assisted activation/carbonisation entry excluded from this evidence class: natural organic acid-mediated surface-confined covalent esterification is a promising and broadly applicable green hydrophobic modification pathway.

**Table 1:** Esterification performance of representative natural organic acids for hydrophobic modification of biomass-derived porous carbons, supporting the surface-confined covalent esterification strategy proposed in this study. Revised: the malic acid/corncob entry (acid activation followed by high-temperature pyrolysis, 500 → 900 °C) is classified separately as “acid-assisted activation/carbonisation”; it proceeds via dehydration/cross-linking and aromatisation and should not be counted as evidence for covalent ester grafting.

Natural Acid	Carbon Precursor	Reaction Type	Key Outcome	Contact Angle (deg)	Adsorption Capacity	Reference
Myristic acid	Sugarcane bagasse porous carbon	Esterification (covalent grafting)	AC-MA: optimal surface-confined covalent esterification esterification paradigm	54 → 134.8	Toluene: 488 mg g^−1^ @ 80% RH	[35]
Citric acid	Water hyacinth biochar	Esterification (FTIR/XPS confirmed)	–COOH introduction via ester linkage	Increased	Methylene blue: 395 mg g^−1^	[37]
Citric acid	Wheat straw biochar	Ammonia + citric acid modification	Surface chemistry altered, ester groups introduced	—	Enhanced VOC adsorption	[42]
Citric acid	Straw biochar (7 wt%, 50 °C, 6 h)	Impregnation + curing	Optimized for hydrophobicity	Hydrophobic	Hymexazol: 9.89 mg g^−1^ (98.9%)	[44]
Malic acid	Corn cob biochar (two-step pyrolysis)	Acid activation + pyrolysis (500 → 900 °C)	hydrophobic (120 deg), new pores Reclassified: acid-assisted activation/carbonisation route—a porosity-development strategy, NOT evidence for surface esterification.	120 (superhydrophobic)	TPH: 1850 mg g^−1^ Kd = 2.77 L/g	[43]
Tartaric/Gallic	Various biomass precursors	Esterification (in-situ conversion)	Theoretical potential (limited data)	—	n.r.	[14]

Table Notes: Data supports the surface-confined covalent esterification strategy proposed in this study. Malic acid entry: reclassified as acid-assisted activation/carbonisation (not esterification evidence).

**Proposition 4.**  *The paradigm shift toward surface-confined covalent esterification prioritizes “moderate hydrophobicity” over extreme water repellency. This approach, which utilizes renewable reagents and mild conditions in accordance with the principles of green chemistry, provides a balanced solution for the selective capture of VOCs in high-humidity environments.*

In 2026, Chen et al. pretreated linden wood biomass with Ganoderma lucidum fungus to form hydrophobic porous biochar (FTBC) with a short-range ordered graphitic structure during pyrolysis [33]. The adsorption mechanism of FTBC for phenolic compounds, extracellular DNA, and nanoplastics shifted from defect-mediated π-π electronic affinity interactions to lattice-driven hydrophobic interactions. The study explicitly states: “Traditional strategies for enhancing the hydrophobicity of carbon-based materials pose significant environmental and economic challenges and conflict with the principles of green chemistry. For example, siloxane grafting often involves the use and release of hazardous chemicals, posing safety risks.” This statement resonates strongly with the paper’s central argument—it validates a feasible pathway for green hydrophobization from the entirely new dimension of “fungal biocatalysis,” providing cross-disciplinary corroboration for the surface-confined covalent esterification paradigm from a synthetic biology perspective.

If we were to stop at the “resolving the trade-off” narrative in Proposition 3, it would be easy to simplify the issue into a linear judgment that “surface-confined covalent esterification is better than exogenous.” However, such simplification not only fails to meet the requirements of dialectical thinking but also cannot accurately reflect the functional demands placed on materials in complex environments. From the perspective of surface wetting theory, greater hydrophobicity is not necessarily better. In applications involving adsorption in aqueous solutions, while extremely hydrophobic or even superhydrophobic surfaces exhibit strong affinity for organic molecules, the material as a whole may fail to be effectively wetted due to its complete repulsion of the aqueous phase. Hamimed and Chatti (2024) explicitly state that the transition from hydrophilic to hydrophobic requires fine-tuning, as activated carbon that is too hydrophobic may encounter mass transfer barriers in water treatment—if water, as the carrier, cannot come into contact with the pore channels, the pollutants dissolved in it cannot be captured [16]. Therefore, for most adsorption processes targeting organic pollutants in water, a state of “moderate hydrophobicity”—where the material as a whole maintains good water wettability while the pore walls exhibit a preference for adsorbing organic molecules—is the most ideal. Surface-confined covalent esterification modification, through the grafting of shorter hydrocarbon chains or aromatic rings, is particularly effective at tuning the surface to this moderately hydrophobic range, whereas exogenous modification tends to create superhydrophobic boundaries with fully fluorinated or long-chain alkyl groups. The difference between the two lies in the trade-off between “adjustability” and “extreme performance,” and more importantly, reflects the choice of “suitability” within specific application contexts.

Furthermore, the dimension of value orientation lends deeper significance to this dialectical relationship. Jha et al. (2021), in discussing surface-modified activated carbon, specifically emphasized that while strong acid oxidation can remove mineral components and enhance hydrophilicity, the toxic byproducts introduced during the process and the highly corrosive conditions themselves undermine the material’s “sustainability.” [12]. Extending this criterion to the field of hydrophobic modification, the perfluorinated compounds involved in fluorination have drawn widespread attention due to their environmental persistence and bioaccumulation; their entire life cycle—from reagent production to waste disposal—is fundamentally at odds with the principles of green chemistry.

Velasco et al. (2021) characterized the water vapor adsorption of fluorinated activated carbon fibers and found the effect to be strongly dose-dependent: mild fluorination (F_2_:N_2_ = 1:9) lowered the BET area by 15.3% (1194 → 1011 m^2^ g^−1^), whereas more aggressive conditions (3:7 and 5:5) lowered it by 49.7% (to 600 m^2^ g^−1^) and 72.3% (to 331 m^2^ g^−1^), with the micropore volume falling from 0.44 to 0.14 cm^3^ g^−1^ across the same series [45]. At fixed partial pressure, raising the treatment temperature to 150 °C changed little (606 vs. 600 m^2^ g^−1^), indicating that the F_2_ partial pressure, rather than the treatment temperature, governs the extent of textural loss. Notably, mild fluorination also enhanced water affinity at low relative humidity, and the authors reported reduced chemical stability for the heavily fluorinated fibres—a reminder that fluorination does not automatically confer hydrophobicity, and that the dose–property relation is itself non-monotonic. This “condition-dependent” characteristic precisely illustrates that the tension between hydrophobicity and structural preservation under the exogenous fluorination paradigm is a contradiction that cannot be fully resolved by process parameters alone. This mechanistic picture is consistent with the persistence and environmental mobility of highly fluorinated organic reagents, whose production, use, and degradation behaviour are comprehensively catalogued in the PFAS literature [46].

Interface-selective adsorption by biomass-based carbon materials has become a cutting-edge research direction in the field of environmental remediation. Tong et al. (2025) proposed the “Carbon Structure Superposition Strategy” (CSSS) for preparing interface-selective biochar for the treatment of hydrophobic organic pollutants and systematically evaluated its full life-cycle environmental benefits from a carbon neutrality perspective [47]. This study marks a shift in the functionalization design of biomass charcoal from mere “structural optimization” to a new phase of “precise regulation of interfacial selectivity.” It also provides strong support from the perspective of carbon structure regulation for the “surface-confined covalent esterification” paradigm proposed in this paper—both point to a core conclusion: the green functionalization of biomass charcoal must be based on a synergistic consideration of “the intrinsic properties of the carbon framework, surface chemical regulation, and full life-cycle environmental impacts.” In their review of sustainable synthetic strategies for converting biomass/biowaste into porous carbon, Nazir et al. (2023) touched upon a key philosophical point: when pursuing functionalization, one should not forget that carbon materials originate from biomass and should retain their sustainable nature as much as possible [48]. Researchers in China are also accelerating their exploration in this direction. Recent research shows that Yang et al. (2026) prepared citric acid-modified straw biochar under conditions of 7% citric acid and 6 h of modification at 50 °C, achieving an adsorption capacity of 9.89 mg g^−1^ for hymexazol and an adsorption efficiency as high as 98.9% [44]. From the perspective of high-value utilization of agricultural and forestry waste, this study provides empirical support from China for the green modification of biochar using natural organic acids, and also echoes the special value of the “surface-confined covalent esterification” paradigm proposed in this paper under the “Dual Carbon” strategy. A full life-cycle assessment of biochar systems further indicates that, under appropriate feedstock and pyrolysis scenarios, biochar production and use can be energetically self-sufficient and climatically favourable [49].

The latest molecular simulation studies provide microscopic evidence for the “structure-chemistry synergy” mechanism. Research by Ma et al. (2025) shows that biochar (VBs) prepared from distillers’ grains as a precursor via chemical activation has a specific surface area ranging from 795.3 to 1716.6 m^2^ g^−1^ and a surface oxygen content exceeding 11%, with adsorption capacities for toluene and chlorobenzene significantly superior to those of commercial activated carbon [50]; Molecular simulations further reveal that when the pore size distribution is around 0.98 nm, acid anhydride functionalization can increase the adsorption capacities of toluene and chlorobenzene to 10.3 times and 16.6 times that of the original graphite pores, respectively, while reducing the adsorption energies to −55.96 kJ/mol (toluene) and −66.66 kJ/mol (chlorobenzene). The significance of this study lies in the fact that it confirms at the molecular scale that the presence of oxygen-containing functional groups is not, in itself, the inevitable source of hydrophilicity—the key lies in whether these functional groups can be “reprogrammed” through chemical transformation into selective adsorption sites for VOCs. This provides profound mechanistic support for the surface-confined covalent esterification paradigm: the esterification reaction is essentially a process of “functional group reprogramming,” converting originally hydrophilic oxygen-containing functional groups into ester-based structures that possess both anchoring and hydrophobic functions. Jha et al. (2021), in turn, distilled this concept into a holistic requirement for the environmental remediation applications of biomass-derived nanomaterials: renewability and high efficiency must be equally prioritized [12].

Mechanistic decomposition of aromatic VOC uptake: The uptake of toluene, benzene, and chlorobenzene on hydrophobic porous carbons is not purely hydrophobic; it reflects several concurrent contributions: (i) hydrophobic/pore-filling partitioning, which dominates at low relative pressure and is enhanced by the removal of surface polar sites and of adsorbed water; (ii) π-π interactions between the aromatic ring and the graphitic domains of the carbon, modulated by the surface O/C ratio; (iii) electrostatic and polar contributions, including the quadrupole moment of benzene and the dipole moment of chlorobenzene, as well as specific interactions with residual heteroatom functionalities; and (iv) capillary condensation in mesopores at higher relative pressure. Hydrophobic modification therefore acts mainly by removing the competitive adsorption of water rather than by directly strengthening the adsorbate–surface interaction, which is why its benefit is most pronounced under humid conditions. These concurrent contributions are consistent with the adsorbate–adsorbent interaction mechanisms and modification effects summarised in a recent critical review of VOC adsorption by porous materials [24]. Classical studies corroborate this decomposition: the adsorption of benzene and toluene at low concentrations is governed primarily by the volume of narrow micropores and is favoured by a low content of surface oxygen groups [51], while grand-canonical Monte Carlo simulations show that water adsorption on carbons proceeds through a distinct cluster-forming mechanism whose onset depends on the density and arrangement of polar surface sites [52].

Consequently, the priority of surface-confined covalent esterification in the value hierarchy is theoretically justified: it is not only a technical means of achieving hydrophobic functionality but also a practical approach that strikes a carefully considered balance between functionality and sustainability. Exogenous modification may still serve as a supplementary option in specific scenarios requiring extreme hydrophobicity or where the material itself lacks functional groups; however, an irreconcilable tension has emerged between its fundamental chemical logic and the core principles of contemporary green chemistry. Therefore, the paradigm shift from exogenous to surface-confined covalent esterification does not imply that one approach immediately supersedes the other; rather, it marks a shift in the dominant logic of materials design from pure performance extremism toward a “controlled hydrophobization” strategy that incorporates ecological rationality.

Proposition 5. *The applicability of surface-confined covalent esterification depends on the “chemical memory” of the carbon precursor—specifically, the abundance of surface oxygen-containing groups. For highly graphitized or inert substrates lacking reactive sites, composite or exogenous strategies are still required, indicating that the choice of modification pathways is context-dependent.*

The rigor of any theoretical proposition hinges on the conscious definition of its applicability boundaries. The core chemical step in surface-confined covalent esterification modification is the condensation or esterification reaction between carboxyl groups and hydroxyl groups on the carbon surface, which fundamentally determines the high dependence of this paradigm on the chemical state of the carbon surface. The reason why medium-temperature pyrolysis–chemical activation processes, as exemplified by Zhao et al. (2026) and Yang et al. (2023), provide an ideal platform for surface-confined covalent esterification is precisely because these processes deliberately retain high oxygen content and abundant reactive functional groups while constructing a multi-level pore structure [3,4]. However, the situation changes significantly when we turn our attention to porous carbon materials carbonized at higher temperatures or with a higher degree of graphitization. In his discussion on the hydrothermal carbonization of sustainable porous carbon, Alatalo (2016) pointed out that the hydrothermal carbonization process can increase the material’s aromaticity and hydrophobic framework, but it also significantly alters the number and types of surface oxygen-containing functional groups [10]. When the carbonization temperature exceeds 800 °C, reactive functional groups such as carboxyl and hydroxyl groups are extensively removed, and the carbon surface tends to become aromatic and passivated. The broader family of hydrothermally produced carbons indeed spans a wide range of surface polarity and functionality, which directly determines the feasible post-functionalization routes [6,53].

Further research has confirmed the decisive influence of the precursor source on the hydrophilic and hydrophobic properties of activated carbon surfaces. In a recent study, Maimaris et al. (2025) systematically compared a hydrophobic activated carbon host matrix—synthesized from waste coffee grounds via simple pyrolysis and chemical activation—with commercial hydrophilic pine needle-based activated carbon [54]. The results indicated that: Activated carbons prepared from different biomass sources and using different processes exhibit significantly different hydrophilic and hydrophobic properties, and these differences in surface chemistry and polarity directly determine the material’s effectiveness in hydrophilic processes (such as H_2_S adsorption) and hydrophobic processes (such as paraffin loading). This finding provides empirical support for the boundary conditions of Proposition 5 in this paper: the applicability of the surface-confined covalent esterification paradigm is not only limited by the abundance of surface functional groups resulting from carbonization temperature but is, at a deeper level, constrained by the chemical “memory” of the biomass precursor itself—which precisely confirms that the selection of a hydrophobic modification pathway is, in essence, a technical decision-making process deeply influenced by the chemical memory of carbon materials. Process design can likewise deliver intrinsic hydrophobicity: high-temperature carbonization of pomelo peel in the presence of graphene oxide, with in-situ thermal reduction of the oxide, was recently shown to yield lightweight sponge-like biochar combining high hydrophobicity with stable oil–water separation performance [55].

Zhang et al. (2025) point out in their review that, due to their natural porous structure, heteroatom self-doping, and tunable surface chemistry, biomass-derived carbon materials exhibit significant variations in surface chemical properties under different carbonization conditions, which directly determine the feasible pathways for subsequent functionalization [56]. Therefore, the effective implementation of the surface-confined covalent esterification paradigm rests on an implicit theoretical premise: the biomass carbon precursor must possess sufficient “chemical convertibility,” meaning its surface must carry oxygen-containing sites available for esterification reactions. When this premise does not hold—for example, in the case of highly graphitized hydrophobic carbon materials—the surface-confined covalent esterification pathway loses the necessary foundation for chemical reactions. In such cases, if further enhancement of hydrophobicity is still required, exogenous grafting or composite modification strategies will once again come into consideration. The study by Wang et al. (2022) is particularly compelling: for the first time, it utilized a dual chain of evidence—combining experiments with GCMC molecular simulations—to demonstrate that AC-MA modified via surface-confined covalent esterification with natural organic acids exhibits outstanding performance, with a toluene adsorption capacity of 488 mg g^−1^ and a selectivity of 150 at 80% RH, while maintaining stable performance after five regeneration cycles [35]. This study marks a significant green reorientation of the concept of “hydrophobic modification”—a shift from exogenous chemical grafting to surface-confined covalent esterification reactions.

This delineation does not negate the value of the surface-confined covalent esterification paradigm; on the contrary, it elevates our understanding of hydrophobic modification strategies to a contextualized theoretical level. It demonstrates that there is no single “one-size-fits-all” optimal modification scheme; a truly scientific understanding should closely link the choice of modification pathways to the thermochemical history of the biomass precursor. As Wang et al. (2024) pointed out in their review on pore engineering of biomass-derived carbon materials, precisely regulating pore structure parameters (pore size, distribution, and interconnectivity) can significantly influence a material’s performance across different application scenarios [57].

This concept of “precise regulation” reminds us that when examining the evolution from silane fluorination to natural organic acids, we are evaluating not merely the merits of two or more specific technologies, but rather two distinct chemical design logics with different contextual applicability. Understanding this allows us to avoid slipping into an absolutist “technological dichotomy” in research on “green hydrophobic modification,” and instead adopt a dialectical approach toward the boundaries and overlap between different paradigms, thereby reserving theoretical interfaces for potential future hybrid modification paradigms.

Thus, through five intrinsically interconnected propositions, this section has completed a comprehensive logical deduction that begins with the inherent contradictions of hydrophobic modification, proceeds through a comparison of mechanisms between exogenous and surface-confined covalent esterification paradigms, and ultimately arrives at dialectical unity and the delineation of boundaries. These propositions are interlinked; taking the fundamental contradiction of “chemical conversion degree versus structural preservation degree” in biomass porous carbon as the logical starting point, they deduce the parameter-dependent structural trade-off of exogenous modification, further reveal the path by which surface-confined covalent esterification transcends this trade-off and its value foundation, and finally delineate the theoretical boundaries of the paradigms’ applicability from a context-dependent perspective. This chain of reasoning paves the way for the specific comparison and critical examination of the two paradigms in the next section, while also providing a solid logical framework for the final theoretical integration. Table 2 provides a comprehensive comparison of the performance of six categories of hydrophobic modification strategies across five dimensions: contact angle, VOC adsorption at high humidity, IAST selectivity, pore structure effects, and environmental sustainability. It is clear that while silanization (TMCS/PDMS) and fluorination can achieve high contact angles, both come at the cost of varying degrees of pore blockage or structural shrinkage, and exhibit low environmental sustainability; In contrast, myristate esterification (AC-MA) leads comprehensively across three key metrics—contact angle (134.8°), toluene adsorption (488 mg g^−1^ @ 80% RH), and IAST selectivity (150)—while simultaneously maintaining the highest levels of pore structure integrity and environmental sustainability. This comparison provides systematic empirical support for the core thesis of this paper.

Limitations of the contact angle as a hydrophobicity metric for porous materials: The water contact angles compiled in this review are measured on compressed pellets or macroscopic surfaces and therefore reflect a composite response—surface chemistry, roughness, and capillary imbibition into the pellet—rather than the intrinsic wettability of the micropore interior. In micropores, whose dimensions approach the molecular scale, the continuum concept of a contact angle is not strictly valid, and adsorption-based metrics are more appropriate. Complementary, pore-sensitive descriptors include water-vapour adsorption isotherms, water uptake at defined relative humidity, immersion calorimetry and, where available, NMR relaxometry or neutron scattering. We recommend that contact angle be reported together with at least one porosity-aware metric; this recommendation is identified in the Outlook as a standardisation need for the field. Indeed, comparative studies show that contact angle, immersion calorimetry, and water-vapour adsorption probe different aspects of carbon hydrophobicity and need not agree [58], and the wettability of porous substrates is itself governed by composite (Cassie–Baxter-type) behaviour rather than by intrinsic surface chemistry alone [59]. The classical wetting models of Wenzel [60] and of Cassie and Baxter strictly apply to macroscopic, continuum-scale surfaces, and contact angle hysteresis on heterogeneous surfaces further undermines single-number wettability metrics [61]; the physics of capillary condensation and imbibition in confined pore geometries [62] therefore provides the more appropriate description of the interaction of water with the pore interior.

**Table 2:** Studies on the hydrophobic modification of biomass-derived porous carbons, grouped by adsorbate class. Entries are grouped by adsorbate class (VOC vapour; aqueous organic pollutant; oil/organic liquid) rather than by modification strategy, because the biomass precursor, the reagent, the carbonisation and activation history, and the test conditions (phase, temperature, relative humidity, initial concentration) all differ between the primary studies. Cross-group comparison of absolute uptake values is therefore not meaningful, and no ranking of strategies should be inferred from this table. Parameters that were not reported in the primary source are marked “n.r.” and have not been estimated or recalculated.

Entry	Biomass Precursor	Carbonisation T/Time (°C/h) ^f^	Activation Method ^f^	Activation Ratio ^f^	O/C (–) ^f^	BET SSA (m^2^ g^−1^) ^f^	Vmicro (cm^3^ g^−1^) ^f^	Water Uptake (mg g^−1^ or @ RH)	Modification Route	Reagent (Full Name; Carbon no.)	Adsorbate Class (Adsorbate)	Test Conditions (Phase, T, RH, C_0_)	Uptake (mg g^−1^) ^a^	Water CA ^b^ (°)	Pore Effect	Comparability ^c^	Ref.
**Group A—VOC vapour (gas phase)**																	
A1	Sugarcane bagasse–derived porous carbon	600 °C, 90 min (5 °C min^−1^, N_2_) ^g^	KOH activation, 800 °C, 90 min	Carbonisation product: KOH = 1:4 (mass)	0.19 ^h^ (precursor AC: C 78.31, O 19.35 wt%)	2483 → 2267 ^g^	1.05 → 0.93 ^g^	0.07 g g^−1^ (vs. 0.15 for AC) ^g^	Surface-confined covalent esterification (exogenous reagent + covalent grafting)	Myristic acid (tetradecanoic acid, C14:0)	VOC vapour (toluene)	Gas phase; 298 K; 80% RH; C_0_ = 100 ppm; 100 mL min^−1^	488	54 → 134.8	Preserved (ΔSSA 8.7%)	Toluene-matched with A2, but T, RH, C_0_ and flow all differ ^m^	[35]
A2	Coconut shell AC (commercial, Guangdong Hanyan) ^j^	n.r. (commercial AC)	n.r. (commercial; steam activation by supplier)	n.r.	n.r.	861.21 → 794.41 (−7.8%) ^n^	0.3463 → 0.3218 ^n^ (Vtotal 0.5638 → 0.4592)	n.r.	Exogenous coating—silanisation (TEOS pre-hydrolysis + TMCS impregnation)	Trimethylchlorosilane (TMCS) + tetraethyl orthosilicate (TEOS) precursor	VOC vapour (toluene)	Gas phase; 303 K; RH 0/60/90%; C_0_ ≈ 393 ppm; 200 mL min^−1^; 100 mg; Y–N model ^n^	Bare-AC Δsat ≈ −45/−64% at RH 60/90%; TMCS-AC Δsat ≈ −6/−9% at RH 60/90% (vs. dry). At RH 90% TMCS-AC gives 91% higher toluene capacity than Bare-AC (absolute saturated values n.r. in abstract)	111.6 → 143.6	SBET −7.8%, Vmicro −7.1%, Vtotal −18.5% ^n^	Toluene-matched with A1, but T, RH, C_0_ and flow all differ ^n^	[32]
A3	Walnut shell (carbonised + activated)	n.r. (Bare-AC supplied commercially; full prep. in Section 4 of [15])	n.r. (Bare-AC supplied commercially)	n.r.	n.r.	1553.79 (Bare-AC, unmodified); PDMS-AC value n.r. ^o,l^	n.r.	n.r.	Exogenous coating—silanisation (PDMS deposition)	Polydimethylsiloxane (PDMS) coating (cur. var. 200 °C optimum)	VOC vapour (benzene)	Gas phase; RH 0, 30, 60, 90%; T = n.r.; dynamic column, Y–N model	Bare-AC Δsat = −55.9% (RH 0 → 90%); PDMS-AC (200 °C) Δsat = −19.3% (RH 0 → 90%)	≈140 (hydrophobic) ^o^	Structure damage (collapse + blockage of pores more serious at higher modification T)	None ^m^	[15]
**Group B—Aqueous organic pollutant**																	
B1	Water hyacinth biochar	300 °C (slow pyrolysis, N_2_, 5 °C min^−1^, 2 h) ^i^	n.a. (no activation step)	n.a. (no activation step)	n.r.	93.00 → 57.08 ^i^	n.r. (Vtotal 0.1126 → 0.0759 cm^3^ g^−1^) ^i^	n.r.	Surface-confined covalent esterification	Citric acid (2-hydroxypropane-1,2,3-tricarboxylic acid, C6)	Aqueous organic pollutant (methylene blue)	Batch, aqueous; C_0_ = 100–600 mg L^−1^ (isotherm range); T = 298 K (25 °C); natural pH ^i^	q_max = 395 mg g^−1^ (Langmuir); 99.5–372.4 mg g^−1^ across C_0_ 100–600 mg L^−1 i^	~120	Reduced (ΔSBET −38.6%, ΔVtotal −32.6%) ^i^	None ^m^	[37]
B2	Straw (秸秆; species n.r. in abstract)	n.r. (abstract only—full text inaccessible; pyrolysis T not reported in abstract)	n.a. (no activation step)	n.a. (no activation step)	n.r.	n.r. (abstract only)	n.r.	n.r.	Surface-confined covalent esterification (CA impregnation + thermal cross-linking)	Citric acid (C6), 7 wt% solution, 50 °C, 6 h	Aqueous organic pollutant (hymexazol)	Batch, aqueous; C_0_ = n.r. (abstract only); T = 50 °C (modification); pesticide load/release test	9.89 mg g^−1^ (98.9% removal) under optimal 7% CA/50 °C/6 h; cumulative release 59.3%	n.r. (abstract)	n.r. (abstract)	None ^m^	[44] ^k^
B3	Wheat straw (Triticum aestivum; Polish field harvest Sep 2022)	650 °C, 60 min, 20 °C min^−1^ (PRC furnace, Czylok); mass yield 32.51% ^p^	None (pyrolysis only) ^p^	n.r.	n.r.	BC 31.74 → BCC 33.83 → BCN 139.89 m^2^ g^−1^ (raw N_2_ isotherms, File A_1; −69% vs. raw straw 103.40 m^2^ g^−1^ for BC) ^p^	V_micro not directly extracted; V_total BC 0.0213 → BCC 0.0314 → BCN 0.1123 cm^3^ g^−1 p^	n.r.	Surface-confined covalent esterification (BCC: CA; BCN: NH_4_OH) ^p^	BCC = citric acid (C6); BCN = ammonia hydroxide (NH_4_OH) ^p^	Aqueous organic pollutant (enrofloxacin, E) + Ag nanoparticles (Ag-NPs)—NOT VOC ^p^	Batch, aqueous; C_0_ not in abstract; pH = natural (BC ≈ 9.5 alkaline, BCN ≈ 9.8, BCC ≈ 6.5 acidified) ^p^	BCN > BC for E and Ag-NPs (BCN is best adsorbent); numerical q_max n.r. in abstract (data in supplementary) ^p^	n.r.	CA modification slightly increases SSA (+6.6%) and V_total (+47%); NH_4_OH modification strongly increases SSA (+340%) and V_total (+427%) ^p^	None ^m^	[42]
**Group C—Oil/organic liquid (reclassified ^d^)**																	
C1	Corn cob biochar (two-step pyrolysis, 500 → 900 °C)	500 → 900 °C (two-step pyrolysis)	Malic acid activation (acid-assisted) ^d^	n.r.	n.r.	n.r.	n.r. (new pores created ^d^)	n.r.	Acid-assisted activation/carbonisation ^d^ (NOT surface esterification)	Malic acid (hydroxybutanedioic acid, C4)	Oil/organic liquid (total petroleum hydrocarbons)	Batch; Kd = 2.77 L g^−1^	1850 ^e^	120 (superhydrophobic)	New pores created	None ^m^	[43]

Footnotes: ^a^ Uptake values are quoted as given in the primary source; units are mg g^−1^ unless stated otherwise. ^b^ Water contact angle measured on compressed pellets or macroscopic surfaces; a macroscopic, effective quantity reflecting surface chemistry, roughness and capillary imbibition, rather than the intrinsic wettability of the micropore interior (see Section 7.1). ^c^ “Adsorbate-matched” indicates that two entries share the same adsorbate but differ in precursor, activation history, reagent or test conditions; such pairs permit only a partial, qualitative comparison and cannot be used to attribute a difference in uptake to the modification chemistry alone. “Adsorbate- and RH-matched” would additionally require the same relative humidity; no pair of entries in this table meets that stricter criterion (see footnote ^m^). ^d^ Acid activation followed by: acid activation followed by high-temperature pyrolysis (500 → 900 °C) proceeds by dehydration/cross-linking and aromatisation and is mechanistically distinct from surface esterification. It is presented here as a porosity-development strategy and should not be counted as evidence for covalent ester grafting. ^e^ Uptake refers to total petroleum hydrocarbons (TPH), a multicomponent mixture, and is therefore not comparable with the single-component VOC values in Group A. The previously used asterisk is replaced by this footnote. CA = water contact angle; TPH = total petroleum hydrocarbons; Kd = distribution coefficient; ΔSSA = change in BET specific surface area upon modification; Vmicro = micropore volume; n.r. = not reported; n.a. = not applicable. ^f^ Structural and textural parameters are compiled as follows. Values are quoted as given in the primary source; parameters not reported there are marked “n.r.” and have not been estimated or recalculated. The high proportion of “n.r.” entries in these columns is itself a finding: it documents the reporting gap that currently prevents any quantitative structure–performance correlation across studies, and it is the rationale for the minimum reporting standard proposed in Section 7.4. ^g^ Entry A1 is the authors’ own work (Wang et al., Chem. Eng. J. 450 (2022) 138070, Ref. [35]); the preparation, textural and water-uptake data for this entry are taken from that paper and from the first author’s doctoral dissertation (Ref. [63]), which reports the same AC/AC-MA pair. Carbonisation: bagasse heated to 600 °C at 5 °C min^−1^ under N_2_ and held for 90 min; activation: the carbonisation product was ground with KOH at a mass ratio of 1:4, heated to 800 °C at 5 °C min^−1^ under N_2_ and held for 90 min, then washed with 1 M HCl and deionised water and dried at 80 °C. Textural parameters (Table 3-2 of the dissertation) are BET specific surface area 2483 m^2^ g^−1^ (AC) → 2267 m^2^ g^−1^ (AC-MA), total pore volume 1.28 → 1.17 cm^3^ g^−1^ and micropore volume 1.05 → 0.93 cm^3^ g^−1^; the 8.7% loss of BET area matches the value quoted in Ref. [35]. Water uptake is the experimental water capacity of the toluene/water vapour mixture at 298 K (Table 1 of Ref. [35]), 0.15 g g^−1^ for AC and 0.07 g g^−1^ for AC-MA, and is reported in the units of the original source; it is not directly comparable with water-vapour uptake measured at a defined relative humidity. Grafting conditions: carbon-to-acid mass ratio 0.52:1, 65 °C, 18 h, ethanol deposition, with N,N′-dicyclohexylcarbodiimide (20 wt% relative to the acid) as dehydrating agent. ^h^ The elemental composition of the precursor porous carbon (AC) was determined by organic elemental analysis (authors’ own measurements, reported in Ref. [63]); C 78.31 wt%, O 19.353 wt%, H 2.707 wt%, N 0.26 wt%, S 0.1261 wt%; sum 100.8 wt%, within the typical tolerance of elemental analysis; the quoted O/C value is the atomic ratio (0.186) calculated from these mass percentages and is rounded to 0.19. Because the elemental analysis was performed on the unmodified precursor rather than on AC-MA itself, this value characterises the precursor and is not the O/C ratio of the grafted material; it is nevertheless reported because it is the quantity that governs the density of native –OH/–COOH sites available for esterification, and hence the applicability of the route (Proposition 5). The corresponding value for AC-MA was not determined and is therefore not reported. XPS was used only to resolve the relative distribution of carbon and oxygen functionalities (C 1s: O–C=O 12.74% for AC vs. 23.40% for AC-MA; C–C 50.26% vs. 59.84%) and does not yield a bulk O/C ratio. ^i^ Entry B1 (Xu et al., Environ. Sci. Pollut. Res. Int. 23 (2016) 23606–23618, Ref. [37]): the biochar was obtained by slow pyrolysis of water hyacinth at 300 °C under N_2_ with no separate activation step, so the activation columns are reported as “none” and “n.r.” respectively. Textural data are quoted from Table 1 of that paper: BET specific surface area 93.00 m^2^ g^−1^ (WB) → 57.08 m^2^ g^−1^ (CAWB) and total pore volume 0.1126 → 0.0759 cm^3^ g^−1^, corresponding to decreases of 38.6% and 32.6%; mean pore width increased from 4.843 to 5.319 nm. Because this low-surface-area biochar is dominated by meso- and macroporosity, the micropore volume is not reported in the source and is therefore marked “n.r.” here; the total pore volume is given in that column in its place and is explicitly labelled. The Pore-effect entry for B1 has been revised from “slightly modified” to the measured values, and this entry should not be read as a like-for-like comparison with the high-surface-area microporous carbons of Group A. ^j^ Entry A2 (Li et al., Sep. Purif. Technol. 272 (2021) 118659, Ref. [32]): the substrate was a commercial coconut-shell activated carbon; modification proceeded by TEOS hydrolysis (introducing surface –OH) followed by impregnation with 30 wt% TMCS in n-hexane for 24 h. Textural data (SBET, total and micropore volume for the bare, TEOS- and TMCS-treated carbons) are taken from Fig. 6 of that paper and are reported in the corresponding columns (see footnote ^n^); the carbonisation temperature, activation method and activating-agent ratio are not reported in the primary source, because the substrate was supplied as a commercial activated carbon, and are therefore marked “n.r.”. The qualitative pore-effect entry (TEOS hydrolytic polymerisation forming a Si–O–Si network reduces mesopore volume, while TMCS substitution reduces surface area) is retained as the mechanistic interpretation of those values. The relative humidity reported here has been corrected from 80% to 90%, which is the value given in the primary source and in Section 3 of this review; consequently A1 and A2 are matched in adsorbate but not in relative humidity, as noted in the Comparability column. ^k^ Entry B2 is cited from the abstract only; the full text was not accessible, so the carbonization temperature, activation method and activating-agent ratio are marked “n.r.”. ^l^ Entry A3: the quoted SBET of 1553.79 m^2^ g^−1^ is that of the unmodified walnut-shell activated carbon (Bare-AC); the value for the PDMS-coated material was not reported in the source accessible to us and is therefore marked “n.r.” rather than inferred. The entry is retained in Group A because the study does report VOC (benzene) uptake under controlled relative humidity, and because its own text states that the coating damaged the pore structure—a self-reported instance of the exogenous-coating penalty discussed in Section 3. It should be noted that the same study reports a humidity-stability advantage for the PDMS-coated carbon (saturated uptake falling by 19.3% from RH 0 to 90%, against 55.9% for the unmodified carbon); this is a genuine benefit of the coating and is acknowledged as such, but it does not offset the structural penalty or the reagent burden. ^m^ No pair of entries in this table satisfies a fully matched comparison: entries A1 and A2 share the adsorbate (toluene) but differ in every other test parameter—298 K vs. 303 K, 80% vs. 90% RH, 100 vs. ≈393 ppm, and 100 vs. 200 mL min^−1^ (footnote ^n^), and no other pair shares even the adsorbate—the entries span toluene, benzene, methylene blue, hymexazol, enrofloxacin with Ag nanoparticles, and total petroleum hydrocarbons. This is a property of the primary literature rather than of the present compilation: no two of the surveyed studies were carried out on the same precursor, with the same activation history, and at the same humidity. Grouping by adsorbate class is therefore the strongest structure the available data support, and the Comparability column is retained precisely so that each comparison is graded explicitly rather than left to imply a degree of equivalence that the underlying data do not possess. Accordingly no ranking of modification strategies is, or can be, inferred from this table; the mechanistic argument developed in Section 3 and Section 4 remains the basis on which the two paradigms are compared. ^n^ Entry A2 (Li et al., Sep. Purif. Technol. 272 (2021) 118659, Ref. [32]): textural data are taken from Fig. 6 of that paper, which tabulates S_BET 861.21 m^2^ g^−1^ (Bare-AC), 860.94 (AC/TEOS) and 794.41 (AC/TEOS-30%TMCS); total pore volume 0.5638, 0.4583 and 0.4592 cm^3^ g^−1^; micropore volume 0.3463, 0.3459 and 0.3218 cm^3^ g^−1^; and average pore width 2.619, 2.129 and 2.312 nm. Two points deserve emphasis. First, the net losses after the full TEOS + TMCS treatment are modest—7.8% of BET area and 7.1% of micropore volume—because TEOS hydrolysis divides mesopores into micropores and thereby offsets part of the loss, even though total pore volume falls by 18.5%; the authors state that TMCS impregnation acts mainly on the surface. Second, and more importantly, these figures do not support a claim that silanisation damages the pore structure more severely than esterification: the losses are in fact comparable to, and marginally smaller than, those of entry A1 (8.7% and 11.4% respectively). The penalty of this route therefore lies elsewhere, and the primary source is explicit about it: in dry gas (RH 0%) the modified carbon adsorbs around 20% less toluene than the unmodified carbon, and the authors conclude that the method is unsuitable for dry conditions, while regeneration is poor (saturated capacity falling by 36–39% over three cycles, worse than the unmodified carbon). Test conditions differ from those of A1 in every respect except the adsorbate: 303 K rather than 298 K, 90% rather than 80% RH, ≈393 ppm rather than 100 ppm, and 200 rather than 100 mL min^−1^. ^o^ Entry A3 (Li et al., Sep. Purif. Technol. 239 (2020) 116517, Ref. [15]): walnut-shell-derived Bare-AC had SBET 1553.79 m^2^ g^−1^ (ScienceDirect abstract). Modification with PDMS at 200 °C was optimum (collapse and blockage of pores became more serious at higher T). PDMS-AC achieved a water contact angle ≈ 140° (Bare-AC is hydrophilic). ^p^ Entry B3 (Tomczyk et al., Chemosphere 356 (2024) 141916, Ref. [42]; dataset DOI 10.18150/XON02P, File A_1): wheat-straw biochar obtained by pyrolysis 650 °C, 60 min, 20 °C min^−1^; mass yield 32.51%. Adsorbates are enrofloxacin (E) + Ag nanoparticles (Ag-NPs), NOT VOC. N_2_ isotherms (raw data, File A_1) re-computed via BET (0.05–0.30) and Gurvich (P/P_0_ ≈ 0.95): S (raw straw) 103.40 m^2^ g^−1^, V_tot 0.1073 cm^3^ g^−1^; BC 31.74/0.0213; BCC 33.83/0.0314; BCN 139.89/0.1123. BCN is the most effective adsorbent for both E and Ag-NPs. Numerical q_max values are not given in the article and reside in the associated dataset (File A_3 of the deposition cited in Ref. [34]).

## 5. Discussion: The “Chemistry-Space-Green” Trilemma and Beyond

**Figure 4:**
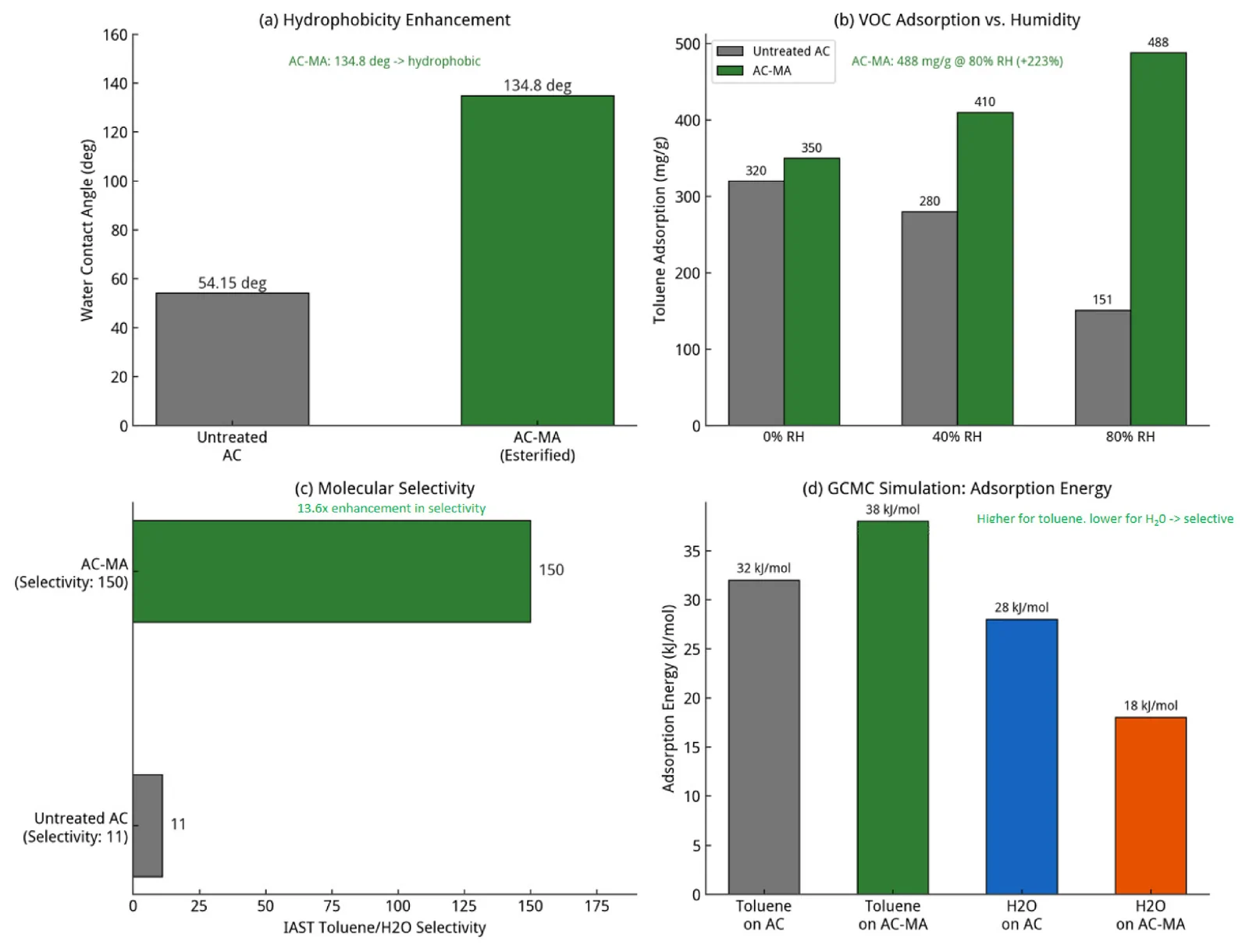
Experimental and computational validation of surface-confined covalent esterification (Wang et al., 2022 [35]). (**a**) Water contact angle: 54.15° → 134.80°. (**b**) Toluene uptake: AC–MA 488 mg g^−1^ at 80% RH (+223%); unmodified AC 320 → 151 mg g^−1^. (**c**) IAST toluene/H_2_O selectivity: 11 → 150 (13.6-fold). (**d**) GCMC adsorption energy: higher for toluene, lower for water on AC–MA. Reproduced with permission.

Figure 4 summarises the key empirical evidence reported by Wang et al. (2022) [35]. Taken together, the four panels show that esterification with myristic acid (C14:0) delivers hydrophobicity, VOC capacity under humid conditions and toluene-over-water selectivity simultaneously, without sacrificing pore structure—the conjunction predicted by the surface-confined covalent esterification paradigm. These data validate the paradigm from both experimental and theoretical perspectives.

In the first two sections, this study established an analytical framework centered on the synergistic optimization of “surface chemical conversion degree and pore structure preservation degree” through a deconstruction of the concept of “green hydrophobic modification” and a logical deduction from the exogenous to the surface-confined covalent esterification paradigm. However, the value of a theoretical framework depends not only on the internal consistency of its logic but also on its ability to engage in effective dialogue with existing academic discourse and to highlight its unique explanatory power while addressing controversies and critiquing limitations. Therefore, this section will place the aforementioned propositions within a broader theoretical context; by identifying areas of consensus, analyzing controversies, and critiquing limitations, it will ultimately distill the innovative perspective of this study.

From the perspective of theoretical consensus, the fundamental conclusions of this study clearly resonate with numerous classic discussions in the fields of materials surface science and green chemistry. First, Proposition 1—which asserts that hydrophobic modification results from the synergistic interaction between surface chemistry and pore structure—is highly consistent with the view emphasized by Li et al. [32] in their review of biomass-based porous carbon for supercapacitors, namely that “surface chemical properties are equally important as pore structure.” That study pointed out that the excellent electrochemical performance of activated carbon stems from the combined contribution of high specific surface area and tunable surface chemical properties, providing theoretical support from a related field for this study’s treatment of “structural integrity” and “chemical conversion” as dual core variables. Second, Proposition 2’s revelation of the pore-blocking effect induced by exogenous modification resonates with the discussion by Hamimed and Chatti (2024) on the water treatment applications of porous carbon [16]. They explicitly point out that the pore characteristics of porous carbon undergo significant changes during the process of transitioning from hydrophilic to hydrophobic through activation, which indirectly confirms the undeniable correlation between hydrophobization and structural damage in exogenous modification pathways. Furthermore, the arguments presented in this study regarding the surface-confined covalent esterification paradigm align with the theoretical orientation conveyed by Yuan et al. (2022) in their research on the construction of microporous biochar via citric acid esterification [14]. That study emphasizes the use of the precursor’s own chemical composition for directed modification, which coincides with the core logic of this study—that “surface-confined covalent transformation is superior to heterogeneous coating.” Moreover, Jha et al.’s (2021) examination of the sustainability of surface-modified activated carbon—in which they noted that while strong acid oxidation can alter surface hydrophilicity, it also introduces toxic byproducts—resonates with the green chemistry evaluation criteria that underpin this study throughout [12]. Together, these findings corroborate the fundamental consensus that the environmental compatibility of modification reagents should be incorporated into the evaluation framework for functional materials. These points of convergence indicate that the theoretical framework of this study is not an isolated academic monologue, but rather a systematic refinement and deepening of core issues built upon the existing research context.

However, the existence of consensus does not imply the elimination of controversy. Current debates within the field regarding the “greening of hydrophobic modification” primarily center on whether modification with natural organic acids can truly replace traditional fluorination and silanization treatments in terms of hydrophobic efficiency. Skeptics often argue that natural organic acids have short hydrocarbon chains and limited hydrophobic efficacy, making it difficult for them to achieve the same hydrophobic effects as fluorocarbon chains; consequently, they lack competitiveness in applications requiring high hydrophobicity. Through the logical deduction of this study’s propositions, it becomes clear that the root of this controversy lies in the differing interpretations of the goal of “hydrophobic modification” itself among the opposing sides. As exemplified by Chandrasekaran et al. (2017)’s research on the evolution of carbon aerogels, studies pursuing extreme wettability often treat superhydrophobicity as the ideal endpoint, naturally leading to a preference for fluorination methods [31]. However, from the perspective of adsorption functionality, as pointed out by Hamimed and Chatti (2024), porous carbon used in water treatment requires a finely tuned hydrophilic-hydrophobic balance rather than an extreme hydrophobic state [16]. Propositions 4 and 5 of this study provide a theoretical pathway to bridge this controversy: in most aqueous VOC adsorption scenarios, while natural organic acid modification does not achieve the same extreme level of hydrophobicity as fluorination, the “moderate hydrophobicity” it achieves falls precisely within the range of greatest practical value for aqueous adsorption. Furthermore, this approach simultaneously ensures good preservation of the pore structure and a fully green process. It is thus evident that the controversy does not stem from the relative merits of the technologies themselves, but rather from differing evaluation criteria—by replacing a single hydrophobicity metric with a three-dimensional, synergistic evaluation system encompassing “chemical transformation, structural preservation, and green value,” the previously seemingly irreconcilable contradiction has gained a new perspective for resolution. This is precisely the clarifying and advancing role that this study plays in the theoretical discourse. From a sustainability standpoint, it is also worth noting that the fluorinated reagents underpinning extreme water repellency belong to the broader class of per- and polyfluoroalkyl substances, whose environmental persistence is now well documented [46]; natural and bio-inspired water-repellent surfaces, by contrast, achieve superhydrophobicity through hierarchical topography rather than fluorinated chemistry [64].

A closer examination of the theoretical limitations in existing research reveals two relatively common shortcomings. First, when discussing the modification of biomass-derived porous carbon, most studies in the existing literature remain confined to method descriptions and performance characterization, lacking a typological distinction between “exogenous” and “surface-confined covalent” modification mechanisms. Although Nazir et al. (2023) conducted a systematic review of sustainable strategies for converting biomass into porous carbon, they failed to distinguish the fundamental similarities and differences between “exogenous coating” and “surface-confined covalent transformation” based on the chemical relationships between the modifying agents and the carbon matrix [48]. Jha et al. (2021) advocated for the sustainable application of biomass-derived nanomaterials in environmental remediation but failed to further abstract surface-confined covalent esterification as an independent, theoretically significant paradigm [12]. As a result, a large body of research on modification using natural organic acids remains at the stage of empirical exploration, and the underlying theoretical significance of “transformation over coating” has not been fully explored. Second, when evaluating modification effects, existing studies generally use end-performance indicators such as contact angle and adsorption capacity as core criteria, while paying little attention to whether the modification process itself complies with the basic principles of green chemistry. While existing review literature has touched upon the tension between functionality and sustainability when analyzing the pore structure of bio-based porous carbon [48], it has not systematically integrated green chemistry principles into the evaluation framework of the modification paradigm. Alatalo (2016) advocated hydrothermal carbonization as a sustainable synthesis route for porous carbon [10]; however, his discussion focused on the carbon scaffold formation stage and did not sufficiently address the environmental impact of the functionalization stage. This has created a theoretical gap: the academic community has failed to provide an analytical framework that organically unifies the chemical logic of modification mechanisms with green value assessments. Consequently, the concept of “green hydrophobization” has either become mere rhetoric or been narrowed down to the simple use of certain natural reagents, failing to rise to the level of a paradigm critique.

Based on this critical examination, this study proposes an innovative analytical perspective that integrates the three dimensions of “chemical logic—spatial effects—green attributes.” The core tenet of this perspective is that the understanding and evaluation of hydrophobic modification of biomass-derived porous carbons cannot be based on a single-dimensional comparison across different pathways but must be systematically examined from three interrelated dimensions. The first dimension is the chemical logic dimension, which examines the chemical reaction mechanisms underlying the modification process—whether it involves exogenous physical adsorption or covalent coating, or surface-confined covalent functional-group transformation and surface restructuring. This dimension determines the nature of the bond and the spatial relationship between the hydrophobic groups and the carbon framework. The second dimension is the spatial effects dimension, which assesses the impact of modification on the geometric integrity of the porous structure—does it lead to microporous blockage and a sharp decline in specific surface area, or does it achieve good preservation of the pore structure? This dimension echoes the issue of “synergy between chemical conversion and structural preservation” raised in Research Proposition 1 of this study. The third dimension is the green attributes dimension, which examines the origin of modification reagents, reaction conditions, byproduct toxicity, and the environmental compatibility of the material throughout its entire life cycle—does it rely on persistent fluorinated synthetic reagents and harsh conditions, or is it based on renewable natural organic compounds and mild esterification reactions? This dimension elevates the sustainability requirements for surface modification emphasized by Jha et al. (2021) from a peripheral concern to an intrinsic evaluation criterion of equal importance to the chemical mechanism [12]. These three dimensions are mutually constraining and defining: chemical logic determines the fundamental characteristics of spatial effects, while green attributes provide a value scale and basis for selection regarding the merits of the chemical logic. Examining the evolution from silane fluorination to natural organic acids through this three-dimensional integrated perspective clearly reveals that this is not merely a simple substitution of hydrophobic reagents, but rather a shift in chemical logic from “heterogeneous coating” to “homogeneous transformation,” in spatial effects from “structural sacrifice” to “structure-friendly,” and in terms of green attributes, from “environmental burden” to “ecological compatibility.” The theoretical advantage of this perspective lies in its ability to transcend the limitations of single performance metrics, simultaneously explaining why natural organic acid modification is inferior to fluorination in terms of hydrophobicity but possesses logical superiority in terms of comprehensive adsorption performance and sustainability; it also explains why, in certain highly graphitized, functionally group-poor carbon materials, the surface-confined covalent esterification pathway is not the optimal choice—because the premise of its chemical logic does not hold, and in such cases, it is necessary to return to a three-dimensional framework for a context-specific re-evaluation.

In summary, this section, through a dialogue with existing theories, has identified a common ground between the logic of surface-confined covalent esterification and classical surface theory as well as the principles of green chemistry; clarified the divergence in evaluation criteria underlying the controversy over the efficiency of hydrophobic modification; and pointed out the theoretical limitations of current research regarding paradigm distinctions and the assessment of green value. The proposal of a three-dimensional integrated perspective—comprising “chemical logic, spatial effects, and green attributes”—serves not only as this study’s response to the aforementioned limitations but also as a theoretical refinement of the prior conceptual deconstruction and propositional deduction. This perspective provides a comprehensive analytical toolkit and critical framework for the systematic theoretical summary presented in the concluding section below, as well as for offering directional recommendations regarding a rational design paradigm for the hydrophobic modification of biomass-derived porous carbon in the future.

Extension to superhydrophobic surfaces: Superhydrophobicity requires the conjunction of low surface energy and hierarchical roughness at two length scales; low surface energy alone is insufficient. Surface-confined covalent esterification readily delivers low surface energy but is a molecular-level, conformal modification that does not by itself generate the required roughness—indeed, it tends to smooth or fill micropore entrances. The concept is therefore best extended to superhydrophobicity in a hybrid architecture: the esterification layer supplies the low-energy chemistry, while hierarchical roughness is supplied either by the intrinsic hierarchical pore network of the biomass-derived carbon or by a subsequent green structuring step (e.g., templating with a bio-derived porogen or renewable wax/particle templates). Because grafting inside micropores works against the Cassie–Baxter state (capillary imbibition draws liquid into the pores), superhydrophobic behaviour is more plausibly achieved on the external surface of carbon particles or on monolithic/coated forms, with the internal porosity reserved for adsorption function. Combining esterification with bio-derived roughness templates is, in our view, the most promising route to a fully green superhydrophobic sorbent; the durability of such hierarchical structures under abrasion remains the main open challenge. The requirement for the conjunction of low surface energy and hierarchical roughness follows from the biological archetype of the lotus effect [65] and from the classical Cassie–Baxter description of wetting on porous, textured surfaces [59].

Tentative structure–performance analysis and descriptor hierarchy for future modelling: A rigorous QSAR is not yet attainable from the available data because the literature reports these systems under non-comparable conditions. On a tentative basis, three observations emerge from the compiled dataset: (1) the retained micropore volume fraction tends to decrease with increasing carbon number of the grafting acid beyond C14–C16, consistent with the steric argument above; data points obtained on mesopore-dominated, low-surface-area biochars should instead be treated as a separate series in terms of total pore volume, since their micropore fractions are not defined on a comparable basis (Table 2); (2) in high-surface-area microporous carbons, polycarboxylic acids (citric, tartaric) appear to preserve microporosity better than long-chain monocarboxylic acids at comparable grafting levels, which we attribute to their smaller cross-sectional footprint and multi-point anchoring. This observation is, however, restricted to microporous hosts. On low-surface-area, mesopore-dominated biochars the same acids can instead produce substantial textural loss, and the direction and magnitude of the change are then governed by pore opening or blocking rather than by the number of carboxyl groups: citric acid modification lowered the BET area of a water-hyacinth biochar from 93.00 to 57.08 m^2^ g^−1^ (−38.6%) and its total pore volume from 0.1126 to 0.0759 cm^3^ g^−1^ (−32.6%) [37], whereas on wheat-straw biochar it raised the area only marginally (31.74 → 33.83 m^2^ g^−1^, +6.6%), the much larger increase to 139.89 m^2^ g^−1^ being obtained with ammonia hydroxide instead [42]; and (3) given the small, heterogeneous dataset, these trends should be read as hypotheses for testing rather than established correlations. For future modelling, we propose a hierarchy of descriptors: geometric (kinetic diameter, cross-sectional area, chain length, degree of branching); energetic (isosteric heat of adsorption, and GCMC-derived adsorption enthalpy for the target VOC); interfacial (surface energy, water contact angle, work of adhesion); and stability (the hydrolysis free-energy barrier). Such descriptor hierarchies are well suited to the data-driven materials discovery workflows that have matured in recent years [66].

Hydrolytic stability and long-term performance of grafted ester linkages: The ester linkage is intrinsically susceptible to hydrolytic cleavage, whereas Si–O–Si networks and especially C–F bonds are considerably more robust (the latter raising well-documented persistence and toxicity concerns). Available evidence indicates that the hydrolysis rate of surface-grafted esters is strongly pH-dependent—base-catalysed hydrolysis is markedly faster than acid-catalysed or neutral hydrolysis—and that hydrophobic local environments and steric shielding near the surface can retard cleavage relative to small-molecule ester analogues. However, systematic, quantitative hydrolysis-kinetics studies on ester-grafted porous carbons under environmentally relevant conditions are essentially missing: to the best of our knowledge, no rate constants or half-lives for such coatings under realistic wastewater pH, temperature, and microbial conditions have been reported. We therefore do not claim long-term durability for this route; its most defensible application window is near-neutral pH, moderate temperature, short-to-medium service cycles, or regenerable/single-use sorbents. Standardised accelerated-aging protocols, pH-dependent kinetic measurements, and assessment of microbially mediated ester cleavage are identified as priority research needs. Direct experimental evidence for the pH-dependence of grafted ester-linkage cleavage—negligible degrafting near neutral pH but measurable cleavage under alkaline, salt-containing conditions—has been reported for ester-linked model coatings [67], underscoring the need for quantitative kinetic data on ester-grafted porous carbons.

**Figure 5:**
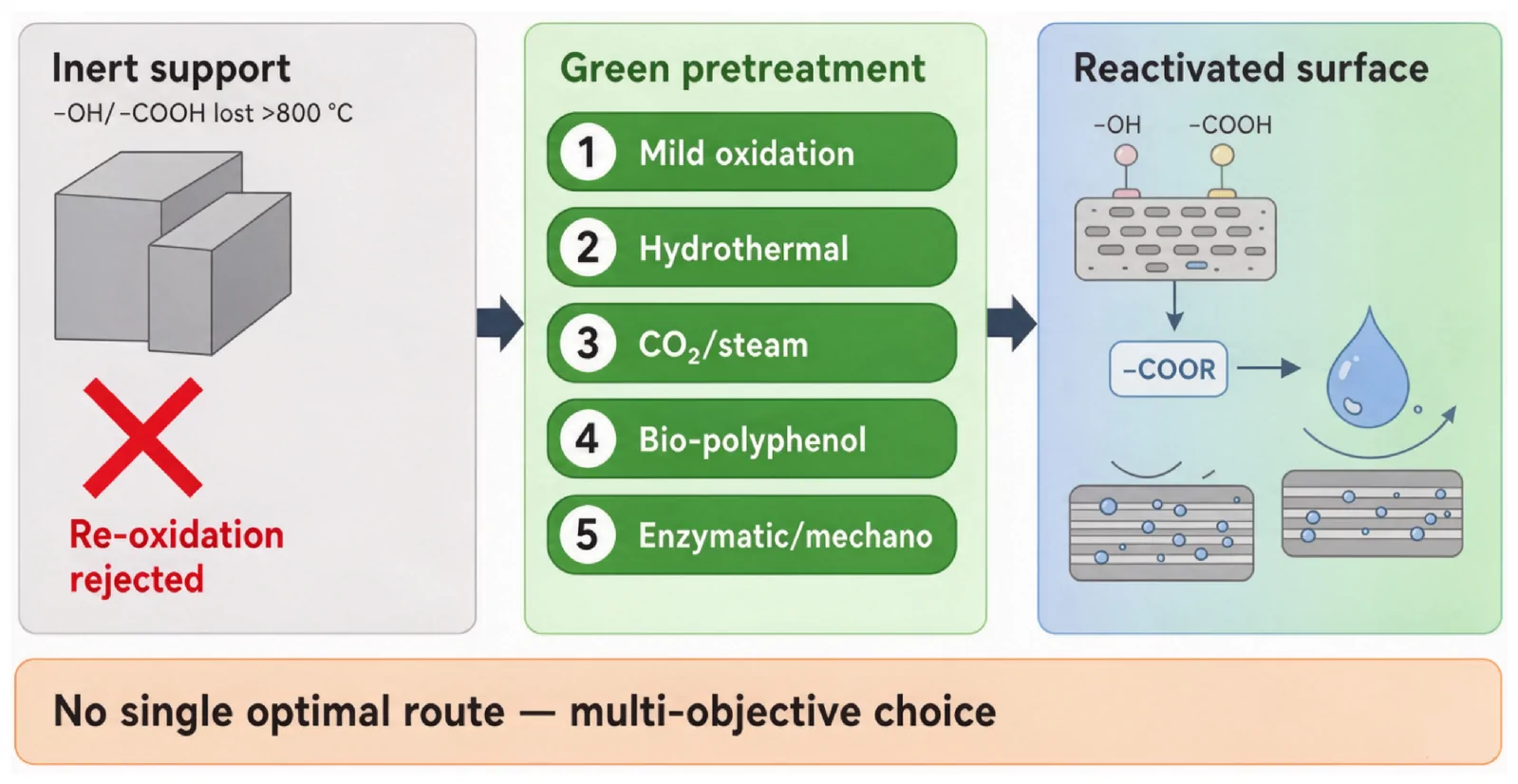
Green hybrid strategy for restoring surface reactivity on inert or highly carbonised supports. Conventional re-oxidation with concentrated mineral acids, dichromate or permanganate is rejected (crossed out) on green-chemistry grounds. Five green pretreatment routes are available: (1) mild oxidation with H_2_O_2_, plasma or ozone; (2) hydrothermal/solvothermal treatment with dilute organic acids; (3) CO_2_ or steam physical activation; (4) bio-derived polyphenol pre-coating; (5) mechanochemical or enzyme-mediated oxidation. Each restores –OH/–COOH anchoring sites at a different energy or cost penalty (Table 3), after which surface-confined covalent esterification yields an application-ready hydrophobic sorbent.

Green hybrid strategy for restoring surface reactivity on inert or highly carbonised supports: Surface-confined covalent esterification presupposes a sufficient density of native –OH/–COOH groups (“chemical memory”), which is largely erased above ~800 °C or after extensive graphitisation. Green pretreatments that can restore surface reactivity without concentrated mineral acids, dichromate/permanganate, or other hazardous oxidants include: (1) mild H_2_O_2_, plasma, or ozone treatment under ambient conditions (water as the only by-product); (2) hydrothermal or solvothermal treatment with dilute organic acids (e.g., citric or acetic acid) as combined activating and functionalising agents; (3) CO_2_ or steam physical activation at moderate temperature, which develops porosity while generating a modest density of oxygen sites; (4) bio-derived polyphenol pre-coating (e.g., tannic acid, lignin, dopamine-analogue layers), which deposits a reactive, –OH-rich interlayer from a renewable source and decouples the grafting chemistry from the underlying carbon’s own functionality; and (5) mechanochemical or enzyme-mediated (laccase/peroxidase) surface oxidation. Each pretreatment imposes its own energy or cost penalty; the selection of a pretreatment is therefore an optimisation problem rather than a universally superior solution. Mild oxidative pretreatments (H_2_O_2_, (NH_4_)_2_S_2_O_8_, or oxygen plasma) are well documented to introduce oxygen functionalities on activated carbons, with plasma producing the highest oxygen loading while largely preserving textural properties [25]; bio-inspired polydopamine coatings can be deposited from aqueous dopamine solutions onto virtually any substrate surface [68], providing readily accessible green entry points for the hybrid strategies outlined above. Figure 5 summarises the resulting hybrid workflow: the five green pretreatment routes restore –OH/–COOH anchoring sites with differing energy and cost penalties (Table 3), after which surface-confined covalent esterification delivers an application-ready hydrophobic sorbent.

## 6. Concluding Remarks

This study focuses on the hydrophobic modification of biomass-derived porous carbon. Centered on the theoretical thread of “a paradigm shift from silane fluorination to natural organic acids,” it constructs a systematic analytical framework through a multidimensional deconstruction of the concept of “green hydrophobic modification,” a progressive deduction of its core propositions, and a critical dialogue with existing theories. The study begins with a clear theoretical inquiry: Has the intrinsic logic of hydrophobic modification of biomass-derived porous carbon undergone a paradigm shift from “exogenous hydrophobization” to “surface-confined covalent restructuring”? How does this shift bridge the theoretical gap between “structural regulation” and “interface design”? Through a step-by-step logical argumentation, this inquiry has been clearly resolved.

The core theoretical findings of this study can be distilled into three progressively elaborated propositions. First, the efficacy of hydrophobic modification of biomass-derived porous carbon is not determined by a single metric—such as contact angle or hydrophobic strength—but is governed by a synergistic optimization relationship between “degree of surface chemical transformation” and “degree of pore structure preservation.” This finding reveals a long-overlooked structural contradiction in the field of hydrophobic modification: the introduction of hydrophobic groups tends to occupy pore space—the extent depending on grafting density and modifier size—creating a tension between chemical conversion and structural preservation that requires careful balancing. Any technical strategy that pursues hydrophobic metrics in a one-dimensional manner may weaken the material’s overall adsorption performance due to structural damage. Second, in the face of this fundamental contradiction, the exogenous modification paradigm and the surface-confined covalent esterification paradigm have adopted two distinctly different approaches to resolution. The exogenous paradigm, represented by silanization and fluorination, involves the chemical coverage or grafting of a heterogeneous hydrophobic layer onto the carbon surface. Consequently, it tends to fall into the “hydrophobicity–pore” trade-off—achieving high hydrophobicity often comes at the cost of (partial) pore constriction or blockage and loss of specific surface area, the severity depending on modifier size, grafting density, and pore size distribution. In contrast, the surface-confined covalent esterification paradigm, based on the esterification of natural organic acids, achieves the surface-confined covalent construction of hydrophobic functionality by converting the carbon surface’s inherent oxygen-containing functional groups into ester structures. This conversion makes the hydrophobic groups a natural extension of the carbon framework rather than a heterogeneous addition, thereby theoretically resolving the contradiction between chemical transformation and structural preservation inherent in the exogenous paradigm. Third, the core value of this paradigm shift lies in its expansion of the evaluation criteria for hydrophobic modification from a single indicator of hydrophobic strength to an integrated assessment system featuring the three-dimensional synergy of “chemical logic—spatial effects—green attributes.” The surface-confined covalent esterification paradigm transcends the exogenous paradigm not only due to its structural compatibility in terms of technical mechanisms but also because of its intrinsic alignment with chemical logic and the principles of green chemistry—it uses renewable natural organic compounds as modifiers and mild esterification reactions as conversion methods, thereby maximizing the preservation of the intrinsic ecological compatibility of biomass materials. This three-dimensional integration has shifted the theoretical discourse on hydrophobic modification from a “efficiency-first” technical narrative to a paradigm narrative that considers “function, structure, and sustainability” in a synergistic manner.

The core theoretical contribution of this study lies in the fact that, for the first time, it classifies the hydrophobic modification of biomass-derived porous carbon into two paradigms—“exogenous coating” and “surface-confined covalent transformation”—and reveals that the former is prone to the “hydrophobicity–porosity” trade-off, with severity depending on modifier size and grafting density due to the formation of heterogeneous layers, while the latter achieves logical consistency through the in situ transformation of functional groups. This typological classification transcends the one-dimensional perspective of previous studies, which treated various modification methods as equivalent, and provides a theoretical framework for green hydrophobic modification that is both explanatory and predictive. First, it corrects the current tendency in the field to “prioritize methods over logic.” Existing studies often treat silanization, fluorination, and modification with natural organic acids as parallel technical options, focusing on the optimization of process parameters and comparisons of end-use performance, yet failing to distinguish them typologically at the level of chemical mechanisms. The “exogenous vs. surface-confined covalent esterification” typological framework proposed in this study provides a clear logical classification standard for disparate modification methods, revealing the fundamental differences between various pathways. Second, it deepens the conceptual understanding of “green hydrophobic modification.” Although the term “green modification” is widely used in academia, its meaning is often vaguely equated with “the use of natural raw materials.” Through conceptual deconstruction, this study strictly defines green hydrophobic modification as a modification paradigm “based on natural, renewable reagents, achieving hydrophobization through the transformation of intrinsic oxygen functionalities, and complying with green chemistry principles throughout the entire process,” thereby providing clear boundaries for the academic use of this concept. Third, this study systematically integrates scattered theoretical insights. Hamimed and Chatti’s (2024) focus on changes in pore characteristics during hydrophobic modification [16], Jha et al.’s (2021) call for sustainable modification [12], and Wang et al.’s (2024) advocacy for precision modification [57]—these insights, previously scattered across different literature sources, have been systematically linked and theoretically elevated within the three-dimensional integrated framework constructed in this study. The value of this framework lies in its provision of a theoretical space capable of simultaneously accommodating analyses of chemical mechanisms, evaluations of structural effects, and assessments of green value, enabling researchers to examine the comprehensive quality of any modification strategy within a clear coordinate system that identifies “which dimension has been sacrificed and which has been optimized.”

In terms of practical implications, although this study focuses on theoretical deliberation, its conclusions offer clear guidance for the rational design of biomass-derived porous carbons. The three-dimensional integrated framework suggests that, when developing novel hydrophobic modification methods, researchers should not rely solely on contact angle or adsorption capacity as single optimization targets, but should simultaneously examine the impact of the modification reaction on pore space and the environmental compatibility of the modification reagents. For porous carbons derived from agricultural and forestry waste such as corn stover and Chinese fir sawdust, the natural organic acid esterification pathway warrants priority attention precisely because these raw materials generally possess abundant surface oxygen-containing functional groups after chemical activation, providing ample chemical sites for surface-confined covalent esterification. At the same time, the limitations of this study serve as a reminder to practitioners: the effective implementation of surface-confined covalent esterification depends on the carbon surface possessing sufficient “chemical convertibility.” For carbon materials subjected to high-temperature graphitization or those lacking functional groups, it may be necessary to explore hybrid strategies combining exogenous and surface-confined covalent esterification, rather than mechanically applying a single paradigm.

This study has clear theoretical limitations and unresolved issues. First, the three-dimensional analytical framework established in this study was primarily developed to address the requirement for “moderate hydrophobicity” in an aqueous adsorption context; its applicability to application scenarios seeking extreme wettability, such as superhydrophobic or self-cleaning surfaces, requires further scrutiny and adjustment. Second, although surface-confined covalent esterification modification logically offers the advantage of structural compatibility, its long-term chemical stability and anti-fouling performance under complex water quality conditions still require systematic experimental validation. Finally, this study focuses on a comparative analysis of mechanisms at the conceptual level. Quantitative structure-activity relationships between the molecular structures of different types of natural organic acids and their modification efficacy still require in-depth experimental exploration and theoretical modeling in future research, guided by the three-dimensional framework. These limitations not only define the scope of this study but also leave open space for further academic dialogue.

## 7. Outlook: Open Questions and Standardisation Needs

The paradigm comparison developed in Section 3, Section 4 and Section 5 rests on concepts—chemical logic, spatial effects and green attributes—that are, at present, only partially operationalised in the primary literature. Before the relative standing of the two paradigms can be established quantitatively, three methodological issues must be resolved: how wettability is measured on porous solids, how the formation of a surface-confined ester linkage is verified, and how long that linkage survives in service. Each is addressed below, together with the data infrastructure required for predictive modelling.

### 7.1. Beyond the Contact Angle: Pore-Sensitive Wettability Metrics

The water contact angles compiled in this review are measured on compressed pellets or on macroscopic surfaces and therefore reflect a composite response—surface chemistry, roughness and capillary imbibition into the pellet—rather than the intrinsic wettability of the micropore interior. In micropores, whose dimensions approach the molecular scale, the continuum concept of a contact angle is not strictly valid, and adsorption-based metrics are more appropriate. Complementary, porosity-aware descriptors include water-vapour adsorption isotherms, water uptake at a defined relative humidity, immersion calorimetry and, where available, NMR relaxometry or neutron scattering. We recommend that any contact angle reported for a hydrophobic porous carbon be accompanied by at least one such descriptor; this is a prerequisite for comparing materials across studies and is proposed here as a standardisation need for the field.

### 7.2. Verification of Surface Ester Formation

Fourier-transform infrared evidence alone is not conclusive for ester formation. Residual carboxylic acid, lactone and adsorbed carboxylate species all contribute to the C=O and O–C=O regions and can be mistaken for an ester. A multi-technique verification protocol is therefore recommended: (i) solid-state ^13^C NMR, in which the ester carbonyl typically resonates at a distinct chemical shift (≈170–175 ppm) from that of free carboxylic acid and carboxylate; (ii) XPS C 1s, in which the ester O–C=O component appears at a higher binding energy (≈288.5–289.0 eV) than the carboxyl/carboxylate component; and (iii) a chemical control, namely base-catalysed hydrolysis of the grafted layer followed by re-measurement of the contact angle and the infrared spectrum, which tests directly whether the spectral feature belongs to a hydrolysable ester. Studies that relied solely on FTIR should be read as providing indicative rather than conclusive evidence, as noted in Section 4/Table 1.

### 7.3. Hydrolytic Stability and Service Lifetime

The ester linkage is intrinsically more susceptible to hydrolytic cleavage than the Si–O–Si networks formed by silanisation or the C–F bonds formed by fluorination, although the latter raise well-documented persistence and toxicity concerns that motivate the search for greener alternatives. Available evidence indicates that the hydrolysis rate of surface-grafted esters is strongly pH-dependent, with base-catalysed hydrolysis markedly faster than acid-catalysed or neutral hydrolysis, and that hydrophobic local environments and steric shielding near the surface can retard cleavage relative to small-molecule ester analogues. We state candidly that systematic, quantitative hydrolysis-kinetics studies on ester-grafted porous carbons under environmentally relevant conditions are essentially missing: to the best of our knowledge, no rate constants or half-lives have been reported for such coatings under realistic wastewater pH, temperature and microbial conditions. Long-term durability is therefore not claimed for this route. Its most defensible application window is near-neutral pH, moderate temperature and short-to-medium service cycles, or regenerable and single-use sorbents. Standardised accelerated-aging protocols, pH-dependent kinetic measurements and assessment of microbially mediated ester cleavage are identified as priority research needs.

### 7.4. Data Infrastructure and Descriptors for Predictive Modelling

A rigorous quantitative structure-activity relationship for the grafting acid is not yet attainable, chiefly because the literature reports these systems under non-comparable conditions. Progress requires a minimum reporting standard: every study of a hydrophobically modified porous carbon should tabulate the biomass precursor, carbonisation temperature and duration, activation method and activating-agent ratio, elemental O/C ratio, BET specific surface area, total and micropore volume, water contact angle, water or water-vapour uptake, and the target adsorbate with its uptake and test conditions, with unreported entries marked “n.r.” rather than estimated. On the modelling side, we propose a hierarchy of descriptors for future work: geometric (kinetic diameter, cross-sectional area, chain length, degree of branching), energetic (isosteric heat of adsorption and GCMC-derived adsorption enthalpy for the target VOC), interfacial (surface energy, water contact angle, work of adhesion) and stability (the hydrolysis free-energy barrier discussed in Section 7.3). A first-generation predictive model is most economically built on GCMC-derived adsorption enthalpy combined with a steric footprint parameter; the tentative trends identified in Section 5 should be read as hypotheses for testing rather than as established correlations.

## Figures and Tables

**Table 3:** Green hybrid pretreatment routes for restoring surface reactivity on inert or highly carbonised supports: typical conditions, oxygen content attained, attainable grafting density, and the principal limitations of each route.

Pretreatment Route	Typical Conditions	O/C Attained (–)	Attainable Grafting Density/Anchoring Sites	Main Limitations and Penalties
(1) Mild oxidation: H_2_O_2_, O_2_/N_2_ plasma, or O_3_	Ambient T and pressure; aqueous H_2_O_2_ (typically 5–30 wt%, 0.5–6 h) or short plasma exposure; no mineral acid	≈0.05–0.15 (plasma gives the highest loading)	Low-to-moderate; introduces –OH, C=O and –COOH directly on the carbon. Plasma largely preserves texture, so the site density gained is not offset by pore loss.	H_2_O_2_/ozone penetrate poorly into narrow micropores; plasma is line-of-sight and difficult to scale into powders; risk of over-oxidation and pore-mouth etching at long exposure.
(2) Hydrothermal/solvothermal with dilute organic acids	180–260 °C, autogenous pressure, 2–12 h; dilute citric or acetic acid (0.1–1 M) as combined activating and functionalising agent	≈0.10–0.25	Moderate-to-high; acid acts as both porogen and functionalising agent, so new sites are created together with new surface.	Thermal energy demand; acid recovery and effluent handling; on low-surface-area, mesopore-dominated biochars the same acids can block pores and lower the BET area substantially (see Table 2, entry B1).
(3) Physical activation: CO_2_ or steam, moderate temperature	700–900 °C under CO_2_ or steam; burn-off typically 20–50 wt%	≈0.02–0.08 (modest by design)	Low; porosity is developed but the oxygen-site density remains limited, so it is best used as a preparatory step before route (1) or (4).	High thermal energy demand and carbon burn-off (yield loss); the oxygen functionality generated is deliberately modest and may still fall below the threshold needed for dense grafting.
(4) Bio-derived polyphenol pre-coating	Aqueous, room temperature, pH ≈ 8.5 (Tris buffer); tannic acid, lignin or dopamine (0.5–5 g L^−1^), 2–24 h	≈0.20–0.40 (within the deposited interlayer)	High; deposits a conformal, –OH-rich interlayer from a renewable source, decoupling the grafting chemistry from the underlying carbon’s own functionality.	Adds a non-carbon layer that occupies pore space; film thickness and uniformity are hard to control inside micropores; long deposition times; precursor cost for purified tannic acid/dopamine.
(5) Mechanochemical or enzyme-mediated oxidation	Ball milling under air/O_2_, or laccase/peroxidase in aqueous buffer at 25–50 °C and near-neutral pH	≈0.05–0.20 (enzyme route highly selective)	Low-to-moderate but highly selective; generates –OH/–COOH with minimal disruption of the bulk texture.	Slow kinetics (hours to days); enzyme cost, stability and the need for mediators; ball milling can collapse microporosity and introduces solid-handling constraints at scale.

^a^ Oxygen-to-carbon atomic ratio attained after pretreatment, as reported for the respective route; the values are indicative ranges compiled from the cited sources and are intended for route selection, not for direct cross-comparison, since the starting O/C of the support differs between studies. ^b^ “Attainable grafting density” is expressed qualitatively because quantitative site densities (sites nm^−2^) are rarely reported; see Section 7.4 on minimum reporting standards. ^c^ Route (1) after Domingo-García et al. [25]; route (4) after Lee et al. [68]. Routes (2), (3) and (5) are described in the green-chemistry and biochar-modification literature cited in Section 5. ^d^ No single route is universally optimal. Each imposes its own energy or cost penalty, and the reachable grafting density differs; the selection of a pretreatment is therefore an optimisation problem rather than the identification of a generally superior solution. Routes may be combined—for example, physical activation followed by mild oxidation, or a polyphenol interlayer applied over a mildly oxidised support.

## Data Availability

No new experimental data were generated for this review; values compiled in Table 2 were extracted from the cited sources, with several entries recomputed from raw isotherm data as indicated in the table notes. The elemental-analysis values reported in footnote ^h^ are the authors’ own measurements and are cited as Ref. [63].
